# Bioeconomic optimization of zinc and iron agronomic biofortification in cowpea (*Vigna unguiculata* [L.] Walp.) in Mozambique under tropical field conditions

**DOI:** 10.1371/journal.pone.0357473

**Published:** 2026-09-03

**Authors:** Tancredo José Carlos, Leonel Domingos Moiana, Omar Jorge Sabbag, Boniface Hussein J. Massawe, Akwilina Wendelin Mwanri, Kallunde Pilly Sibuga

**Affiliations:** 1 Department of Crop Science and Horticulture, College of Agriculture, Sokoine University of Agriculture, Morogoro, Tanzania; 2 Licungo University, Murrópue Campus, Quelimane, Zambézia, Mozambique; 3 Regional Research Center of Leadership for Rice, Instituto de Investigação Agrária de Moçambique, Namacurra, Zambézia, Mozambique; 4 Department of Agriculture and Animal Health, School of Agriculture and Life Sciences, University of South Africa, Roodepoort, South Africa; 5 Department of Phytotechnics, Food Technology and Socioeconomics, São Paulo State University (UNESP), Ilha Solteira, Brazil; 6 Department of Soil and Geological Sciences, College of Agriculture, Sokoine University of Agriculture, Morogoro, Tanzania; 7 Department of Human Nutrition and Consumer Sciences, College of Agriculture, Sokoine University of Agriculture, Morogoro, Tanzania; University of Agriculture Faisalabad, PAKISTAN

## Abstract

Agronomic biofortification with zinc (Zn) and iron (Fe) has emerged as a promising approach to enhance crop productivity while improving food and nutritional security. However, fertilizer recommendations based exclusively on maximum biological yield may not maximize farmers’ economic returns. This study integrated agronomic and economic analyses to identify the biologically and economically optimal Zn and Fe fertilization strategies for cowpea (*Vigna unguiculata* [L.] Walp.) under tropical field conditions in Mozambique. A randomized complete block design with a 3 × 4 × 4 factorial arrangement was used to evaluate three fertilization methods (soil, foliar, and combined soil + foliar) and four application rates of elemental Zn and Fe. Grain yield response was adequately described by a second-order polynomial regression model (R^2^ = 0.767), with maximum technical efficiency predicted at 18.92 kg ha ⁻ ¹ Zn and 19.17 kg ha ⁻ ¹ Fe. In contrast, maximum economic efficiency was achieved at lower application rates (16.94 kg ha ⁻ ¹ Zn and 10.09 kg ha ⁻ ¹ Fe), reducing Fe input by 47.4% while maintaining more than 97% of the maximum predicted grain yield and increasing net profit. Across the evaluated treatments, the combined soil + foliar application produced greater grain yield and agronomic efficiency than either application method alone. Economic robustness was further evaluated through break-even, sensitivity, and stochastic risk analyses, which indicated positive economic performance across the evaluated treatnebts under the agronomic, environmental, and economic conditions considered in this study. Within the agronomic, environmental, and economic conditions evaluated in this single-site, single-season study, the economic optimum was reached before the biological optimum, demonstrating that integrating agronomic and economic optimization can substantially reduce micronutrient inputs while maintaining high productivity and profitability. These findings provide an evidence-based framework for developing economically efficient Zn and Fe fertilization strategies for cowpea under tropical smallholder farming systems.

## Introduction

Cowpea (*Vigna unguiculata* (L.) Walp.) is one of the most important grain legumes cultivated in tropical and subtropical regions, particularly in Sub-Saharan Africa (SSA), where it plays a key role in food security, household income, and sustainable agricultural production [1,2]. The crop is widely grown by smallholder farmers because of its remarkable adaptation to drought-prone environments, short growth cycle, and ability to fix atmospheric nitrogen, thereby improving soil fertility while reducing dependence on synthetic nitrogen fertilizers [1,3]. In addition to its agronomic importance, cowpea grains constitute an important source of dietary protein, vitamins, and essential minerals for millions of people whose diets rely heavily on plant-based foods [4,5].

Despite its importance, cowpea productivity in many tropical production systems remains considerably below its genetic potential. Among the major constraints, deficiencies of essential micronutrients, particularly zinc (Zn) and iron (Fe), are common in highly weathered tropical soils characterized by low organic matter content, poor nutrient retention, and limited micronutrient availability [6]. These nutritional limitations reduce plant growth, impair physiological processes associated with biomass accumulation and reproductive development, and decrease nutrient-use efficiency, ultimately reducing grain yield and farm profitability [6,7]. Similar micronutrient constraints have been widely reported for tropical agricultural systems across Sub-Saharan Africa, emphasizing the need for locally adapted nutrient management strategies.

Micronutrient deficiencies in agricultural soils are closely linked to human nutrition. Zinc and iron deficiencies are among the leading causes of hidden hunger worldwide, affecting more than two billion people and contributing to impaired immune function, anemia, reduced cognitive development, and increased health risks, particularly among women and children in low-income countries [8,9]. Previous research further emphasized that adequate dietary Fe and Zn are essential for cognitive development, neurological function, healthy growth, and overall human well-being, reinforcing the importance of increasing the micronutrient density of staple foods through sustainable agricultural strategies [10]. Therefore, improving both the productivity and nutritional quality of staple crops has become a major component of global food and nutrition security initiatives.

Agronomic biofortification, based on the application of mineral fertilizers containing essential micronutrients, has emerged as a practical and rapidly deployable strategy for simultaneously improving crop performance and increasing the concentration of micronutrients in edible plant tissues [7,11,12]. Unlike conventional breeding, agronomic biofortification can be implemented immediately within existing production systems and therefore provides rapid benefits to farmers while complementing long-term genetic improvement programs. Younas et al. (2025) showed that integrated nitrogen and iron fertilization can improve grain Fe concentration and bioavailability while maintaining agronomic performance in bread wheat. Numerous studies have reported positive effects of Zn and Fe fertilization on crop growth, grain yield, nutrient-use efficiency, and grain micronutrient concentration [9,14,15].Likewise, Alkarawi [16] reported that the combined application of Fe- and Zn-based fertilizers significantly improved vegetative growth, nutrient uptake, biomass production, and grain yield in soybean, demonstrating the complementary physiological roles of these micronutrients in legume productivity. Nevertheless, crop responses remain highly dependent on fertilizer source, application method, nutrient interactions, soil characteristics, and environmental conditions, highlighting the importance of developing site-specific fertilizer recommendations.

Recent studies have further demonstrated that balanced micronutrient nutrition enhances several physiological processes associated with crop productivity, including photosynthesis, antioxidant defense, enzyme activity, and nutrient metabolism. For example, Muhae-Ud-Din et al. [17] reported that micronutrient-based nanofertilizers improved photosynthetic performance, antioxidant enzyme activity, nitrogen metabolism, seed quality, and grain yield, providing further evidence that integrated micronutrient management promotes multiple physiological processes involved in crop productivity. Although the magnitude of these responses varies among crops and production environments, these findings collectively reinforce the importance of balanced Zn and Fe nutrition for sustainable crop production.

Although the agronomic benefits of Zn and Fe fertilization are well documented, fertilizer recommendations are still predominantly based on maximizing biological yield. For smallholder farmers, however, fertilizer management decisions depend not only on productivity but also on production costs, expected economic returns, and investment risks. This consideration was particularly relevant in the present study because Fe-EDTA was substantially more expensive than the Zn fertilizer source and therefore represented a major component of the total micronutrient fertilization cost. Younas et al. [13] emphasized that fertilizer management should simultaneously consider agronomic performance and grain nutritional quality to maximize the practical benefits of biofortification programs. However, fertilizer rates that maximize biological productivity do not necessarily maximize economic return when input costs and crop prices are explicitly considered [18].

The distinction between Maximum Technical Efficiency (MTE) and Maximum Economic Efficiency (MEE) has therefore become increasingly important for developing economically viable fertilizer recommendations. While MTE identifies the nutrient combination that maximizes crop yield, MEE identifies the combination that maximizes economic return by simultaneously considering crop response and production costs [18]. Despite its practical importance, relatively few studies have integrated agronomic and economic optimization to establish micronutrient fertilizer recommendations for grain legumes cultivated under tropical field conditions.

Response Surface Methodology (RSM) provides a robust analytical framework for evaluating nonlinear crop responses, nutrient interactions, and optimization criteria simultaneously. By integrating biological and economic response functions, RSM allows the identification of fertilizer combinations that maximize both productivity and profitability, providing more realistic recommendations for smallholder farming systems than yield-based approaches alone [19]. Furthermore, response surface models enable the quantification of nutrient interactions and the estimation of biologically and economically optimal fertilizer rates, thereby supporting evidence-based nutrient management strategies under contrasting production conditions.

Therefore, this study aimed to integrate agronomic and economic analyses to identify the biologically and economically optimal combinations of Zn and Fe fertilization for cowpea production under tropical field conditions in Mozambique using Response Surface Methodology (RSM). Specifically, the study sought to quantify the divergence between Maximum Technical Efficiency (MTE) and Maximum Economic Efficiency (MEE) and to identify fertilization strategies that maximize farm profitability while maintaining high grain productivity and promoting more sustainable micronutrient management for smallholder farming systems.

## Materials and methods

### Study area description

**Fig 1:**
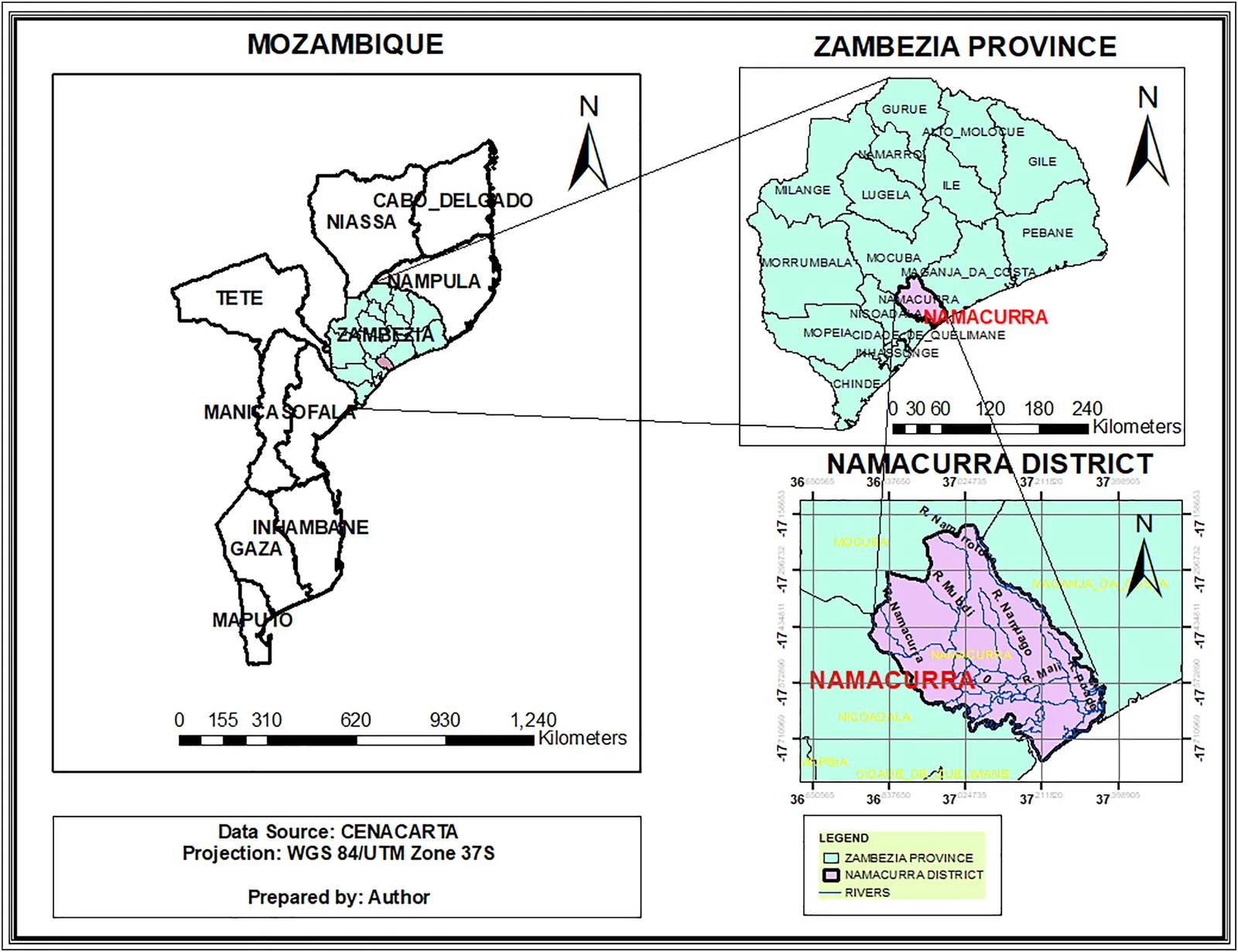
Location of the experimental site at the Instituto de Investigação Agrária de Moçambique (IIAM), Namacurra, Zambézia Province, Mozambique.

The field experiment was conducted during the 2025 rainy season at the experimental station of the Instituto de Investigação Agrária de Moçambique (IIAM), located in Namacurra District, Zambezia Province, Mozambique (17°19′29″ S, 37°01′00″ E; 35 m above sea level) (Fig 1).

**Fig 2:**
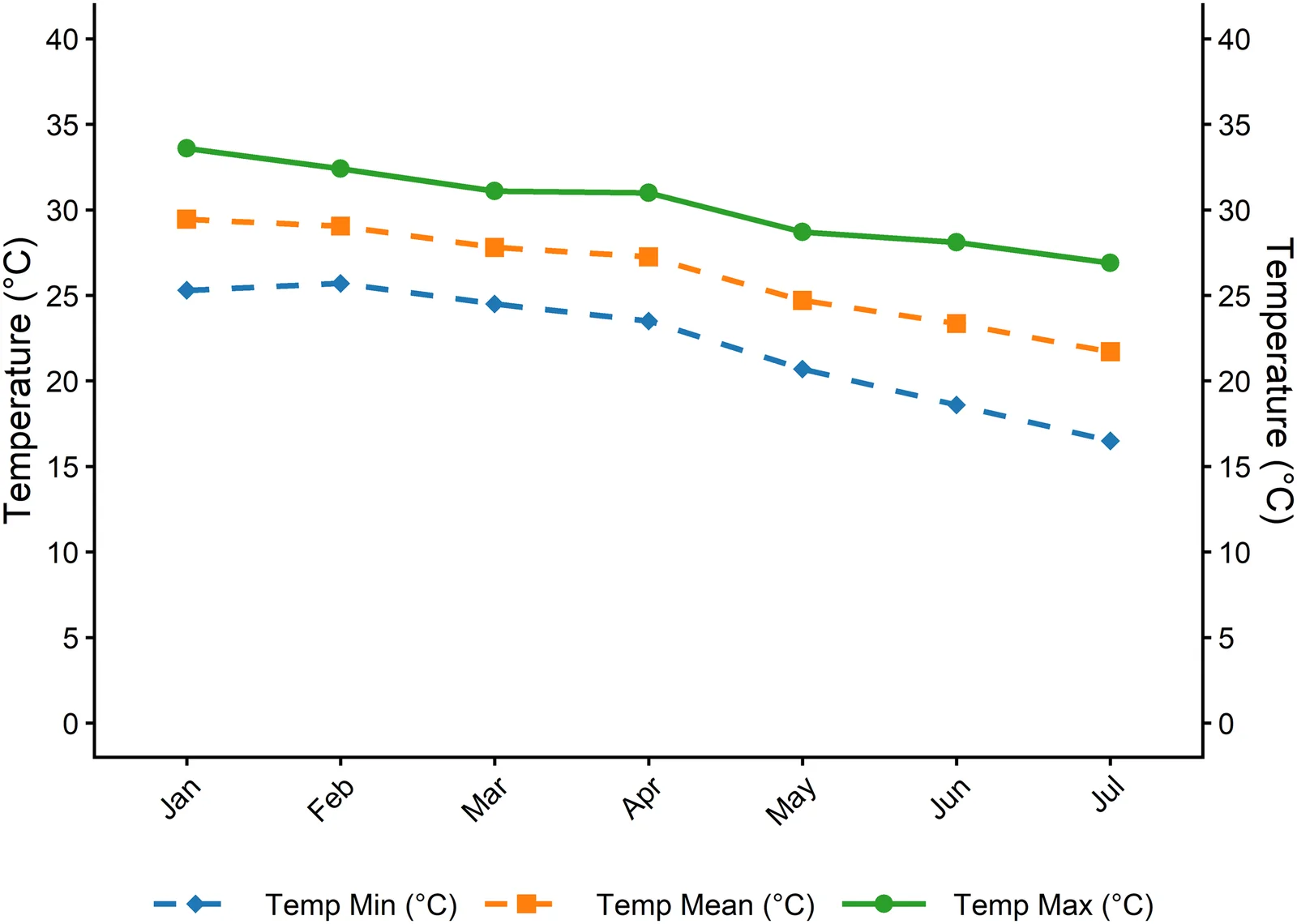
Monthly variation of maximum and minimum air temperature (°C) during the cowpea growing season.

**Fig 3:**
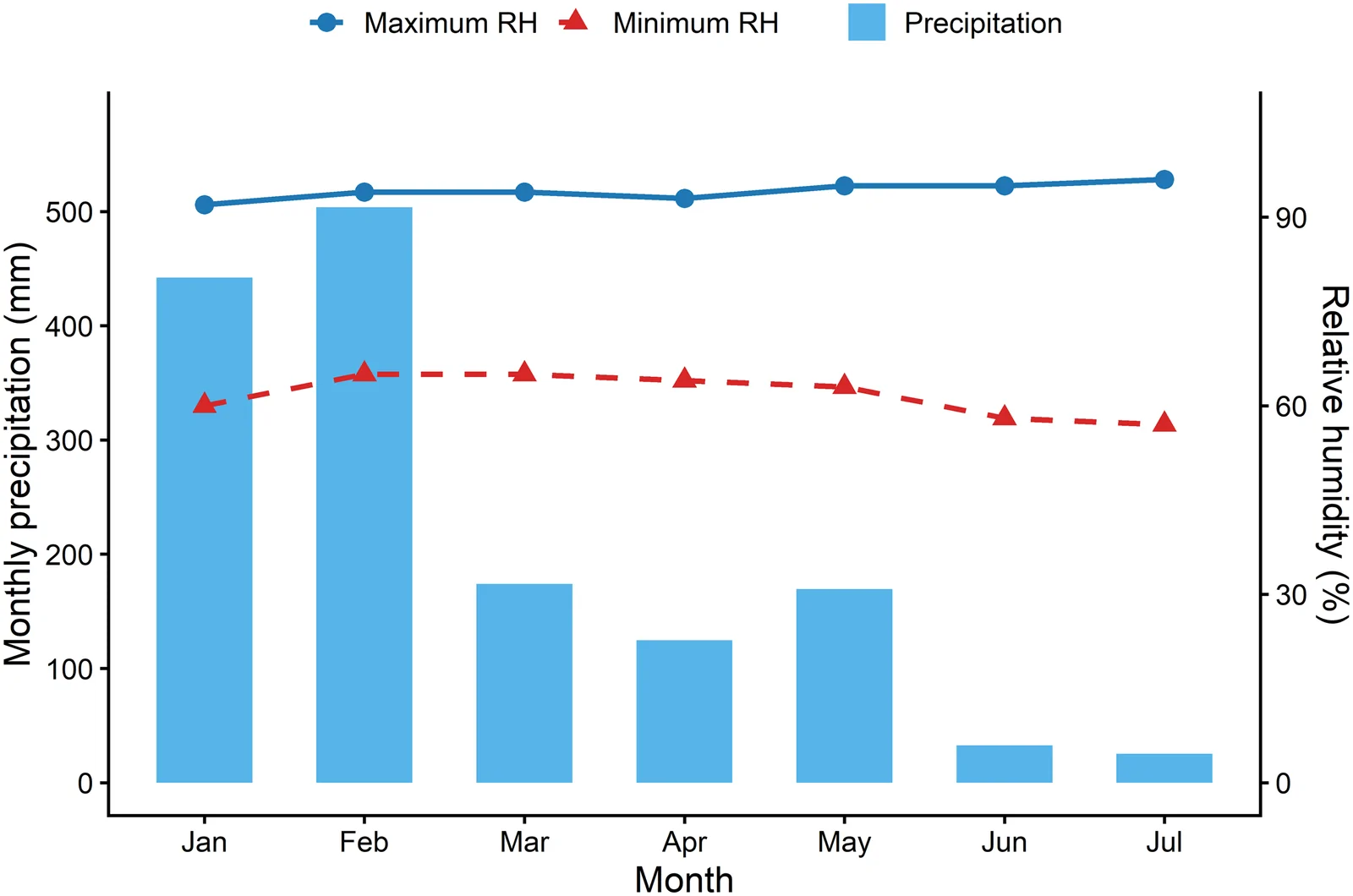
Monthly variation of relative humidity (%) and total precipitation (mm) during the cowpea growing season.

According to the Köppen–Geiger climate classification, the study area has a tropical savanna climate (Aw), characterized by a warm rainy season and a distinct dry season. Meteorological conditions were monitored throughout the crop cycle using data from the Quelimane meteorological station, the nearest station to the experimental site. During the growing season, the mean maximum and minimum air temperatures were 30.18 and 22.11 °C, respectively. Relative humidity ranged from 62% to 94%, while cumulative rainfall reached 1,471.8 mm. Monthly variations in air temperature, rainfall, and relative humidity during the experimental period are presented in Figs 2 and 3. To assess whether the 2025 growing season was climatically representative of the region, monthly mean temperature and rainfall during the crop cycle (April–July) were compared with the long-term climate normals of the Quelimane meteorological station.

### Soil Analysis

Prior to crop establishment, composite soil samples were collected from the 0–30 cm soil layer to characterize the initial physicochemical properties of the experimental area. Sampling was performed using a Dutch auger following a systematic zigzag pattern to obtain representative samples across the field. Soil analyses were conducted according to standard laboratory procedures. Organic carbon was determined by the Walkley–Black method, total nitrogen by the Kjeldahl method, available phosphorus by the Bray I extraction method, exchangeable potassium by ammonium acetate (NH₄OAc) extraction, available sulfur by CaCl₂ extraction, and available Zn and Fe by DTPA extraction.

The experimental soil had a sandy-loam texture with a pH of 6.9. Chemical analysis indicated low concentrations of organic carbon (<17 g kg ⁻ ¹), available phosphorus (<2.0 mg kg ⁻ ¹), and DTPA-extractable Zn (0.1 mg kg ⁻ ¹), indicating Zn deficiency. In contrast, exchangeable potassium (41 mg kg ⁻ ¹) and DTPA-extractable Fe (31.0 mg kg ⁻ ¹) were considered adequate, whereas total nitrogen ranged from 1.0 to 2.0 g kg ⁻ ¹. According to the National Soil Map of Mozambique, the study region is predominantly characterized by Arenosols, Ferralsols, Lixisols, and Acrisols. However, no site-specific pedological survey or WRB/USDA soil classification was available for the experimental field. Therefore, the soil was characterized based on its measured texture and physicochemical properties. The complete physicochemical characterization of the soil, together with the analytical methods used, is presented in Table 1.

**Table 1:** Initial physicochemical characteristics of the experimental soil (0–30 cm depth).

Soil property	Unit	Value	Analytical method
Texture class	—	Sandy loam	Hydrometer method
pH (H₂O)	—	6.9	Potentiometric
Organic carbon	g kg ⁻ ¹	<17	Walkley–Black
Total nitrogen	g kg ⁻ ¹	1–2	Kjeldahl
Available phosphorus	mg kg ⁻ ¹	<2.0	Bray I
Available sulfur	mg kg ⁻ ¹	5.0	CaCl₂ extraction
Exchangeable potassium	mg kg ⁻ ¹	41	NH₄OAc extraction
DTPA-extractable Zn	mg kg ⁻ ¹	0.1	DTPA extraction
DTPA-extractable Fe	mg kg ⁻ ¹	31.0	DTPA extraction

### Experimental design and treatments

The experiment was established using a randomized complete block design (RCBD) in a 3 × 4 × 4 factorial arrangement, with four replications, resulting in a total of 192 experimental plots. Treatments were randomly allocated within each block using a table of random numbers to minimize the influence of spatial variability across the experimental area [20].

Three experimental factors were evaluated: (i) fertilization mode, consisting of soil application, foliar application, and combined soil plus foliar application; (ii) zinc fertilization, applied as zinc sulfate (ZnSO₄·H₂O, 35% Zn) at rates of 0, 7.5, 15.0, and 22.5 kg Zn ha ⁻ ¹; and (iii) iron fertilization, applied as Fe–EDTA (13% Fe) at rates of 0, 5.0, 15.0, and 25.0 kg Fe ha ⁻ ¹.

The selected Zn and Fe application rates were established based on the initial soil fertility assessment and previous agronomic recommendations for micronutrient management under tropical conditions. The range of application rates was designed to correct the identified Zn deficiency while capturing crop responses across agronomically relevant fertilizer levels, thereby enabling the estimation of both Maximum Technical Efficiency (MTE) and Maximum Economic Efficiency (MEE) using Response Surface Methodology.

### Crop establishment and management

The experimental area was prepared by conventional tillage, consisting of plowing followed by harrowing to provide a uniform seedbed for crop establishment. Meteorological conditions were monitored throughout the experimental period to characterize the environmental conditions under which the study was conducted. Because the experiment was carried out under rainfed tropical conditions, soil moisture was not continuously monitored. However, rainfall distribution was recorded during the growing season, and supplemental irrigation was applied during the pod development stage whenever necessary to prevent water stress and ensure adequate grain filling.

Each experimental plot consisted of 11 rows, each 3.4 m long, spaced 0.30 m apart, with 0.20 m between plants within the row, resulting in a total plot area of 5.95 m^2^. To minimize border effects, grain yield and all agronomic measurements were obtained from the net plot area (4.02 m^2^), which excluded the two outer rows and 0.30 m from both ends of each row.

Cowpea (*Vigna unguiculata* [L.] Walp.) cultivar IT-16 was manually sown on 12 April 2025. Two seeds were placed per planting hole at a depth of approximately 2 cm. Basal fertilization was applied at sowing according to the results of the pre-planting soil analysis and consisted of 155 kg ha ⁻ ¹ monoammonium phosphate (MAP), 40 kg ha ⁻ ¹ muriate of potash (MOP), and 5 kg ha ⁻ ¹ urea, ensuring that macronutrient supply was not limiting during crop development.

Micronutrient treatments consisted of zinc supplied as zinc sulfate monohydrate (ZnSO₄·H₂O, 35% Zn) and iron supplied as Fe–EDTA (13% Fe). Soil applications were incorporated into the planting furrows at sowing, whereas foliar applications were divided into two equal applications at 40 and 45 days after planting (DAP), corresponding to the beginning (R1) and full flowering stages, respectively. This application schedule was adopted to synchronize micronutrient supply with periods of high physiological demand [15].

Micronutrient solutions were prepared using deionized water to avoid ionic interference and applied with a calibrated manual backpack sprayer. All foliar applications were performed during the late afternoon to reduce evaporative losses and minimize the risk of leaf injury under high solar radiation [21]. Weed control and thinning were performed manually at 30 DAP to maintain a uniform stand of two plants per planting hole throughout the experiment.

Plants were harvested manually at physiological maturity following the standardized sampling procedures recommended by the HarvestPlus program for agronomic biofortification studies [22]. Grain yield was determined from the net plot area and subsequently converted to kilograms per hectare. To ensure comparability among treatments, grain yield was standardized to 14% moisture content before statistical and economic analyses.

### Economic analysis

The economic performance of Zn and Fe agronomic biofortification was evaluated under field conditions representative of tropical cowpea production in Mozambique. To facilitate international comparison and interpretation, all production costs originally recorded in Mozambican Metical (MZN) were converted to United States dollars (USD) using the prevailing exchange rate during the 2025 cropping season.

The baseline production cost (Cbase) included all standard agronomic practices required for cowpea cultivation, including land preparation, seed purchase, basal fertilization, crop establishment, field management, harvesting, and post-harvest operations, excluding micronutrient fertilization. The baseline production cost was estimated at USD 234.72 ha ⁻ ¹, based on production records and operational cost estimates provided by the Instituto de Investigação Agrária de Moçambique (IIAM), where the field experiment was conducted. The use of IIAM records ensured that the economic evaluation reflected production conditions and management practices representative of experimental cowpea production under tropical conditions in Mozambique. This cost excluded all expenses associated with Zn and Fe fertilization, which were calculated separately according to the fertilizer source, application rate, and fertilization method for each treatment.

The additional cost of micronutrient fertilization was calculated separately for zinc and iron according to fertilizer source, application rate, and market price. Zinc fertilizer cost (CZn) and iron fertilizer cost (CFe) were estimated as:


$$\begin{array}{c} CZn=DoseZn\times \text{0}.\text{8}\text{7} \end{array}$$ (1)

$$\begin{array}{c} CFe=DoseFe\times \text{5}.\text{6}\text{3} \end{array}$$ (2)

where DoseZn and DoseFe represent the application rates of zinc sulfate and Fe–EDTA (kg ha ⁻ ¹), respectively. Fertilizer prices were obtained from commercial agricultural suppliers in Mozambique during the 2025 cropping season, ensuring that the economic analysis reflected the prevailing market conditions during the experimental period.

Application costs (Capp) included labor and operational expenses associated with each fertilization strategy. These costs varied according to the application method and were estimated at USD 43.82 ha ⁻ ¹ for soil application, USD 62.59 ha ⁻ ¹ for foliar application, and USD 106.41 ha ⁻ ¹ for the combined soil plus foliar application.

The total production cost (TC) for each treatment was calculated as:


$$\begin{array}{c} TC=\;Cbase+CZn+CFe+Capp \end{array}$$ (3)

Gross revenue (GR) was calculated by multiplying the corrected grain yield by the average farm-gate price of cowpea grain:


$$\begin{array}{c} GR=\;Yield\;\times Price \end{array}$$ (4)

where grain yield was expressed in kg ha ⁻ ¹ at 14% moisture content, and the grain selling price was fixed at USD 0.70 kg ⁻ ¹, corresponding to the average farm-gate price observed in local markets during the 2025 harvest period.

Net profit (NP) and the benefit–cost ratio (B:C) were then calculated using the following expressions:


$$\begin{array}{c} NP=GR-TC \end{array}$$ (5)

$$\begin{array}{c} B:C=\frac{GR}{TC} \end{array}$$ (6)

where NP represents the economic return after deducting total production costs from gross revenue, whereas B:C expresses the economic return generated per unit of production cost. Treatments with B:C > 1 were considered economically feasible, indicating that gross revenue exceeded total production costs. Together, Net Profit and the Benefit: Cost ratio provide complementary indicators of economic performance by quantifying both the absolute financial return and the economic efficiency of fertilizer investment.

To evaluate the robustness of the proposed fertilization strategies under variable production and market conditions, complementary break-even, sensitivity, and stochastic risk analyses were performed using the observed experimental dataset. Break-even analysis was used to determine the minimum grain yield required to offset production costs, sensitivity analysis quantified the relative influence of the principal economic variables on net profit, and stochastic risk analysis estimated the probability of obtaining positive economic returns under the evaluated production scenarios.

### Statistical analysis and response surface optimization

Grain yield data were first subjected to analysis of variance (ANOVA) according to a randomized complete block design arranged in a 3 × 4 × 4 factorial scheme, with three fertilization modes, four Zn application rates, and four Fe application rates. The statistical model included the effects of block, fertilization mode, Zn rate, Fe rate, and all possible interactions among the treatment factors. When significant treatment effects were detected by the F-test (P < 0.05), means for fertilization modes were compared using Tukey’s honestly significant difference (HSD) test at the 5% probability level.


$$\begin{array}{c} Y_{ijkl\;}=\mu +B_{i\;}+M_{j}+Z_{k}+F_{l}+{\left(MZ\right)}_{jk}+({MF)}_{jl}+(ZF{)}_{kl}+(MZF{)}_{jkl\;}+\varepsilon ijkl \end{array}$$ (7)

where Y_ijkl_ is the observed response; μ is the overall mean; B_i_ is the block effect; M_j_ is the fertilization mode effect; Z_k_ is the zinc dose effect; F_l_ is the iron dose effect, and εijkl is the experimental error.

Prior to statistical analysis, model assumptions were evaluated by examining residual normality and homogeneity of variances. Normality was assessed using the Shapiro–Wilk test, whereas homogeneity of variances was evaluated using Levene’s test. Residual plots were also visually inspected to verify model adequacy before proceeding with subsequent analyses. When significant treatment effects were detected by the F-test (p < 0.05**),** comparisons among fertilization modes were performed using Tukey’s honestly significant difference (HSD) test at the 5% probability level.

To quantify the combined effects of Zn and Fe fertilization on grain yield, response surface methodology (RSM) was applied by fitting a second-order polynomial regression model including linear, quadratic, and interaction terms:


$$\begin{array}{c} Y=\beta_{\text{0}}+\beta_{\text{1}}Zn+\beta_{\text{2}}{Zn}^{\text{2}}+\beta_{\text{3}}Fe+\beta_{\text{4}}{Fe}^{\text{2}}+\beta_{\text{5}}(Zn\times Fe) \end{array}$$ (8)

where *Y* represents grain yield (kg ha ⁻ ¹), and Zn and Fe correspond to the applied fertilizer rates (kg ha ⁻ ¹). Model adequacy was evaluated using the coefficient of determination (R^2^), adjusted coefficient of determination (adjusted R^2^), statistical significance of regression coefficients, and residual diagnostics.

The maximum technical efficiency (MTE) was determined from the stationary point of the fitted response surface, corresponding to the nutrient combination that maximized predicted grain yield within the experimental domain.

To complement the agronomic evaluation, economic performance was assessed using two derived indicators: net profit (NP) and benefit–cost ratio (B:C), both calculated from grain yield and treatment-specific production costs. These variables were analyzed separately using the same factorial ANOVA model adopted for grain yield to evaluate the effects of fertilization mode, Zn rate, Fe rate, and their interactions.

Subsequently, a second-order polynomial regression model was fitted using net profit as the response variable to determine the economically optimal combination of Zn and Fe application rates.


$$\begin{array}{c} NP=\beta_{\text{0}}+\beta_{\text{1}}Zn+\beta_{\text{2}}{Zn}^{\text{2}}+\beta_{\text{3}}Fe+\beta_{\text{4}}{Fe}^{\text{2}}+\beta_{\text{5}}(Zn\times Fe) \end{array}$$ (9)

where *NP* is expressed in USD ha ⁻ ¹. Model adequacy was evaluated using the coefficient of determination (R^2^), adjusted coefficient of determination (adjusted R^2^), statistical significance of the regression coefficients, and residual diagnostics. The stationary point of the fitted response surface was used to estimate the maximum economic efficiency (MEE), defined as the combination of Zn and Fe application rates that maximized net economic return.

Agronomic efficiency (AE) of Zn and Fe fertilization was calculated as the increase in grain yield per unit of nutrient applied according to the following equation:


$$\begin{array}{c} AE=\;\frac{Y_{f}-Y_{\text{0}}}{Nutrient\;applied} \end{array}$$ (10)

where *Yf* is the grain yield obtained under fertilized conditions, *Y0* is the grain yield of the corresponding unfertilized control treatment, and *Nutrient applied* represents the amount of Zn or Fe applied (kg ha ⁻ ¹). Agronomic efficiency was expressed as kilograms of grain produced per kilogram of nutrient applied (kg grain kg ⁻ ¹ nutrient).

All statistical analyses, analysis of variance, regression modelling, response surface optimization, and graphical visualization were performed using R software (version 4.5.2; R Core Team, 2025). Statistical significance was declared at P < 0.05 throughout the study.

## Results

### Climatic conditions during the experimental season

The climatic conditions observed during the 2025 cowpea growing season were generally representative of the regional climate, although rainfall distribution differed from the long-term average in some months (Table 2). Rainfall during May was substantially higher than the historical average, whereas June and July were slightly drier than normal. Mean air temperature remained close to the long-term climatic normals throughout the experimental period, indicating that the experiment was conducted under conditions broadly representative of the local production environment.

**Table 2:** Comparison between the climatic conditions observed during the 2025 cowpea growing season and the long-term climate normals at Quelimane Meteorological Station.

Month	Mean temperature normal (°C)	Mean temperature 2025 (°C)	Rainfall normal (mm)	Rainfall 2025 (mm)	Interpretation
April	25.9	27.25	124.6	124.7	Typical
May	23.9	24.70	61.5	169.4	Wetter than average
June	22.0	23.35	40.1	32.7	Slightly drier than average
July	21.5	21.70	52.7	25.3	Drier than average

**Source:** Experimental weather data recorded at the Quelimane Meteorological Station during the 2025 growing season and long-term climate normals obtained from the Japan Meteorological Agency (JMA).

### Analysis of variance for grain yield

Analysis of variance showed that cowpea grain yield was significantly affected by block, fertilization mode, Zn application rate, Fe application rate, and all interaction terms among the experimental factors (P < 0.001; Table 3). The significant interactions indicate that the effects of Zn and Fe on grain yield depended on the fertilization mode, demonstrating that the responses to micronutrient application were not independent. The significant block effect confirms that the randomized complete block design effectively accounted for spatial variability across the experimental area. Overall, grain yield was strongly influenced by both the main effects of fertilization mode and micronutrient application rates and by their interactions.

**Table 3:** Analysis of variance for cowpea grain yield as affected by fertilization mode, zinc (Zn) rate, iron (Fe) rate, and their interactions.

Source of variation	df	SS	MS	F	P
Block	3	16,632.49	5,544.16	365.14	<0.001
Fertilization mode (M)	2	576,142.53	288,071.26	18,972.71	<0.001
Zinc rate (Zn)	3	2,456,502.23	818,834.08	53,929.37	<0.001
Iron rate (Fe)	3	499,419.15	166,473.05	10,964.11	<0.001
M × Zn	6	95,889.82	15,981.64	1,052.57	<0.001
M × Fe	6	21,576.92	3,596.15	236.85	<0.001
Zn × Fe	9	152,749.41	16,972.16	1,117.81	<0.001
M × Zn × Fe	18	15,286.84	849.27	55.93	<0.001
Residual error	141	2,140.87	15.18	—	—
Total	191	3,836,340.24	—	—	—

**Notes:** df, degrees of freedom; SS, sum of squares; MS, mean square; F, F statistic; M, fertilization mode; Zn, zinc application rate; Fe, iron application rate. The residual mean square (MS error) was used as the denominator for all F-tests.

### Response surface model for grain yield

The combined effects of Zn and Fe application rates on cowpea grain yield were adequately described by a second-order polynomial regression model (Table 4). The fitted equation was:

**Table 4:** Estimated coefficients of the second-order polynomial regression model describing the response of cowpea grain yield to zinc (Zn) and iron (Fe) fertilization.

Parameter	Estimate	p-value
Intercept	1473.68	<0.001
Zn	29.15	<0.001
Zn^2^	−0.90	<0.001
Fe	13.20	<0.001
Fe^2^	−0.47	<0.001
Zn x Fe	0.256	<0.001

**Notes:** Zn, zinc application rate (kg ha ⁻ ¹); Fe, iron application rate (kg ha ⁻ ¹). The fitted model included linear, quadratic, and interaction terms. Positive coefficients indicate an increase in grain yield with increasing nutrient application, whereas negative quadratic coefficients indicate diminishing returns at higher application rates.


$$\begin{array}{c} \text{Y}=\text{1473}.\text{68}+\text{29}.\text{15Zn}-\text{0}.\text{90Z}{\text{n}}^{\text{2}}+\text{13}.\text{20Fe}-\text{0}.\text{47F}{\text{e}}^{\text{2}}+\text{0}.\text{256}(\text{Zn}\times \text{Fe}) \end{array}$$ (11)

All linear, quadratic, and interaction terms were statistically significant (P < 0.001; Table 4), demonstrating that both micronutrients contributed significantly to grain yield variation within the experimental range. The model explained 76.7% of the total variation in grain yield (R^2^ = 0.767), with an adjusted coefficient of determination (adjusted R^2^) of 0.761, indicating good predictive performance under the experimental conditions.

The positive linear coefficients for Zn and Fe indicate that grain yield increased with increasing micronutrient supply at lower application rates, whereas the negative quadratic coefficients demonstrate diminishing marginal responses as fertilizer rates increased. In addition, the positive Zn × Fe interaction coefficient indicates that the simultaneous application of both micronutrients produced a synergistic effect on grain productivity. The fitted response surface was subsequently used to estimate the stationary point corresponding to the maximum technical efficiency (MTE), as presented in the following section.

### Maximum technical efficiency (MTE)

Optimization of the fitted response surface identified an interior stationary point corresponding to maximum technical efficiency. The predicted maximum grain yield was 1,875.97 kg ha ⁻ ¹ at application rates of 18.92 kg Zn ha ⁻ ¹ and 19.17 kg Fe ha ⁻ ¹. Because these optimum rates were within the experimental range, the estimated maximum was obtained by interpolation, supporting the biological interpretation of the fitted model.

**Fig 4:**
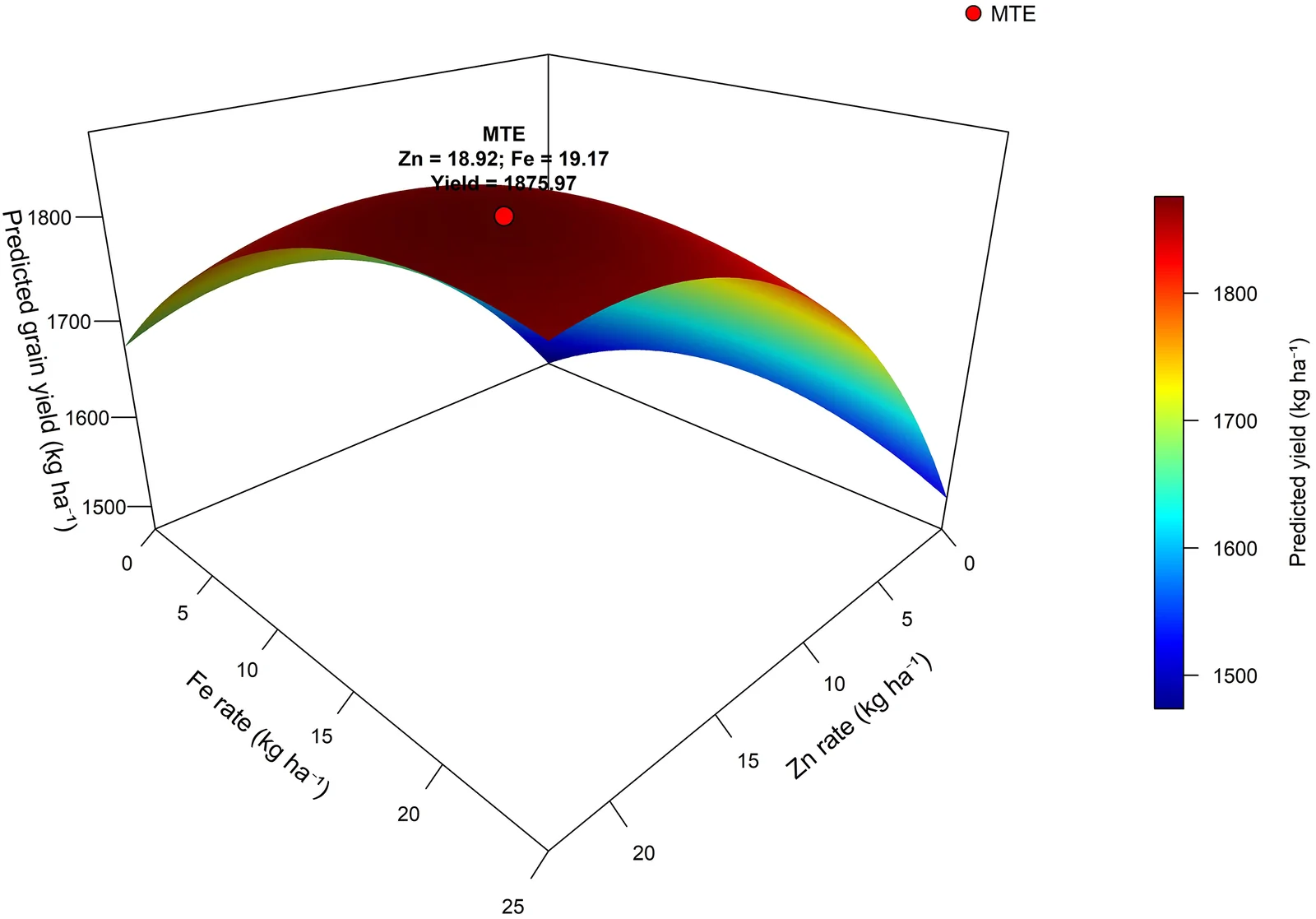
Response surface for the combined effects of zinc and iron application rates on predicted cowpea grain yield. The marker indicates the maximum technical efficiency, estimated at 18.92 kg Zn ha ⁻ ¹ and 19.17 kg Fe ha ⁻ ¹, corresponding to a predicted grain yield of 1,875.97 kg ha ⁻ ¹.

**Fig 5:**
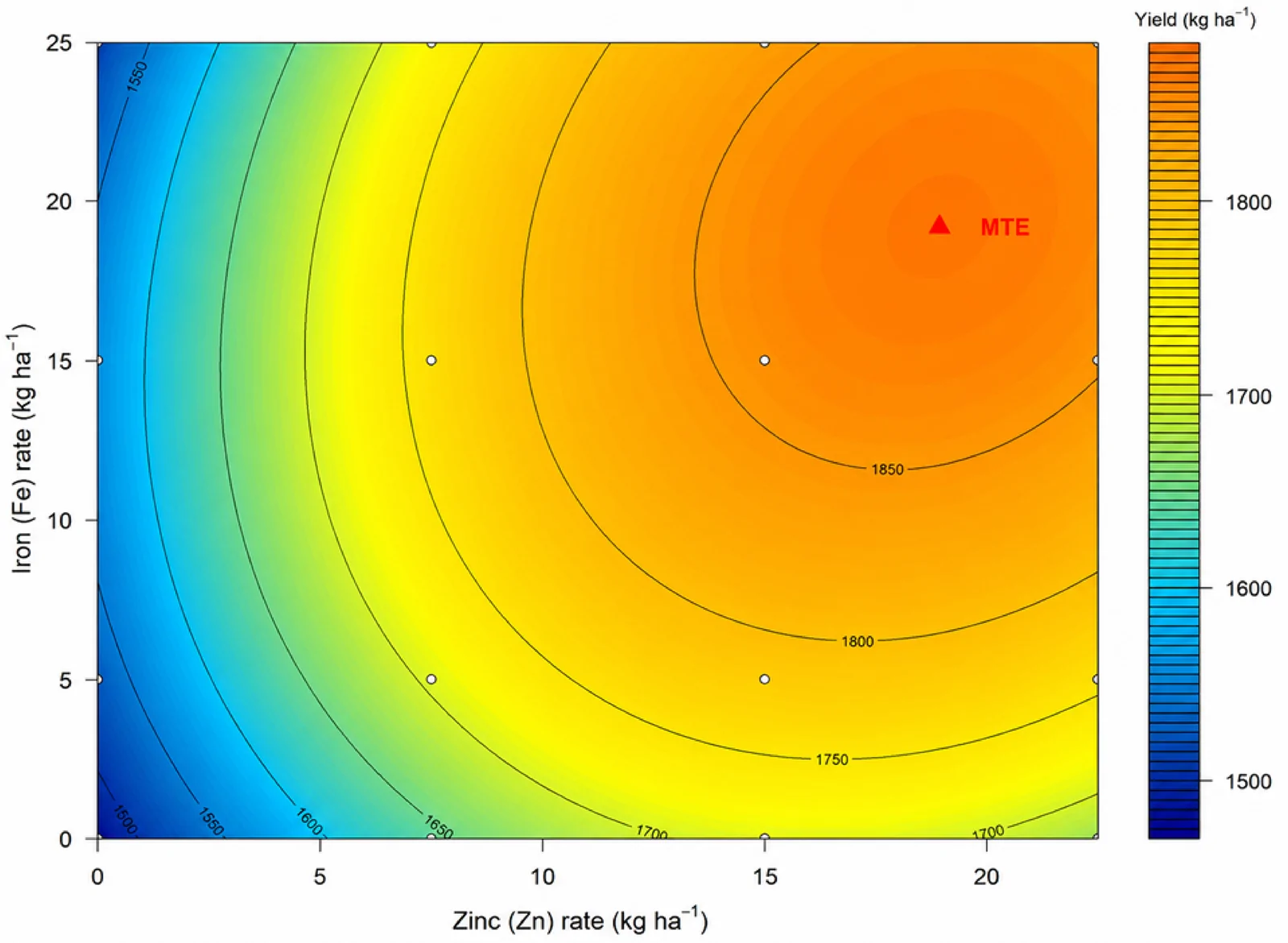
Contour plot of predicted cowpea grain yield across zinc and iron application rates. The red triangle indicates the estimated point of maximum technical efficiency (18.92 kg Zn ha ⁻ ¹ and 19.17 kg Fe ha ⁻ ¹), corresponding to a predicted grain yield of 1,875.97 kg ha ⁻ ¹.

Predicted grain yield increased progressively with increasing Zn and Fe application rates, reaching a maximum near the stationary point before declining at higher nutrient rates. This response pattern and the well-defined optimum region are illustrated by the response surface and contour plot (Figs 4 and 5).

### Effect of fertilization mode

**Fig 6:**
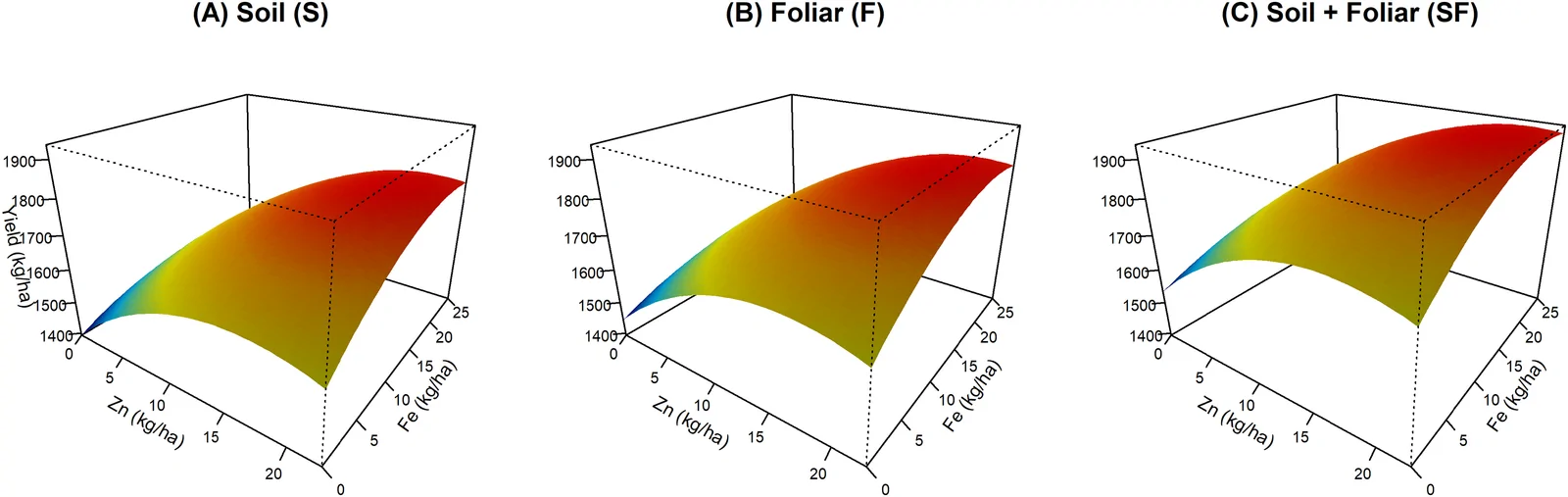
Response surface of cowpea grain yield under different fertilization modes (soil, foliar, and soil + foliar).

Response surface analysis revealed clear differences in predicted grain yield among fertilization strategies. The combined soil + foliar application consistently produced the highest predicted yields, outperforming both soil-only and foliar-only fertilization (Fig 6). These results indicate greater nutrient-use efficiency when both application pathways were combined.

### Agronomic efficiency of zinc and iron

**Fig 7:**
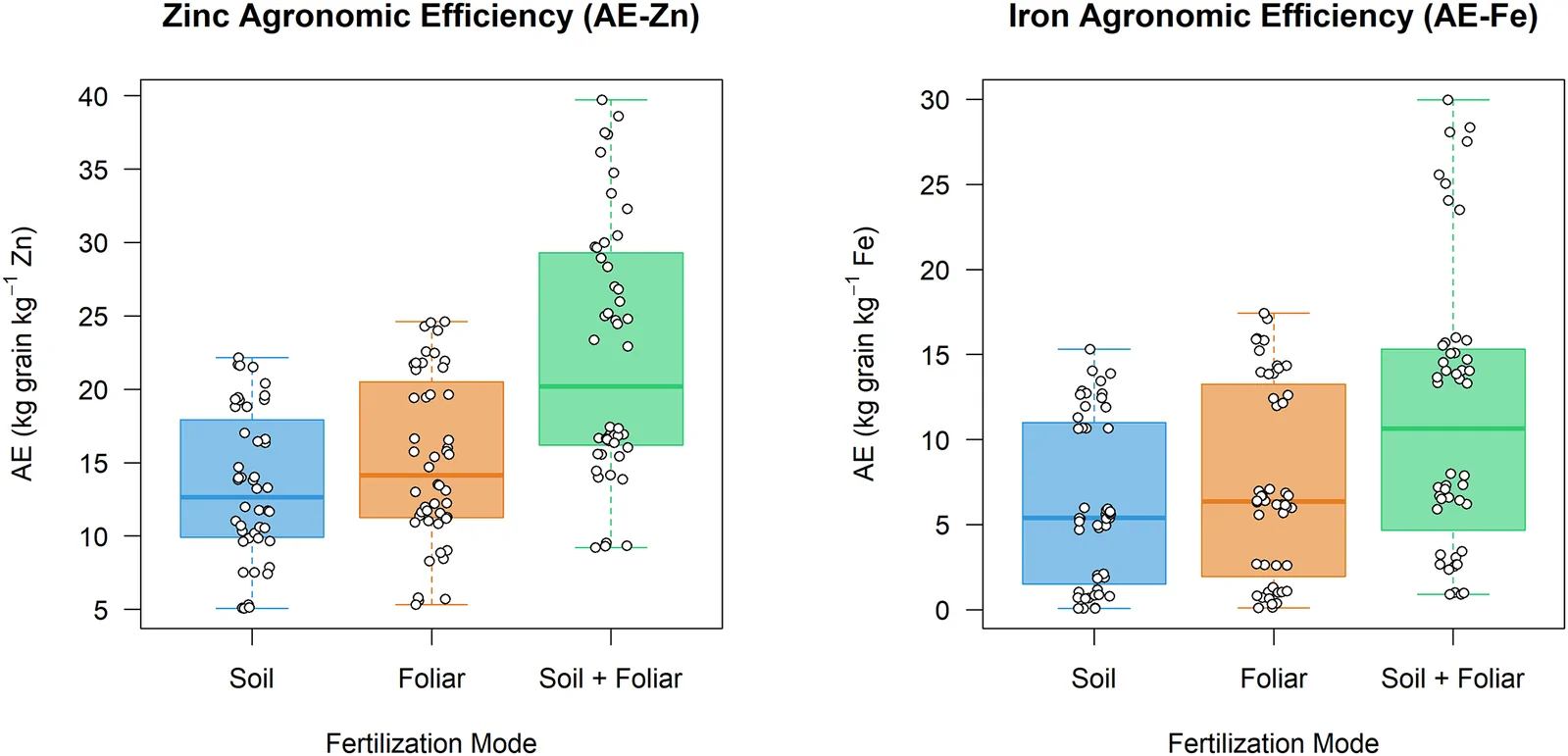
Agronomic efficiency of zinc (Zn) and iron (Fe) under different fertilization modes.

Agronomic efficiency differed among fertilization modes for both Zn and Fe (Fig 7). In both cases, the combined soil + foliar strategy produced the highest agronomic efficiency, followed by foliar-only and soil-only application. Mean AE-Zn values were 13.3, 15.1, and 22.4 kg grain per kg Zn applied for soil, foliar, and soil + foliar application, respectively. Corresponding AE-Fe values were 6.24, 7.34, and 11.5 kg grain per kg Fe applied. These findings indicate that integrating soil and foliar fertilization enhanced nutrient use efficiency and its conversion into grain yield under field conditions.

### Analysis of variance of economic indicators

Analysis of variance showed that fertilization mode, Zn rate, Fe rate, and all interaction terms significantly affected both the benefit–cost ratio (B:C) and net profit (NP) (Table 5). Block effects were also significant, indicating some spatial variation in economic performance across the experimental area. However, the substantially larger F-values associated with fertilization mode and micronutrient application rates indicate that treatment effects greatly exceeded block-to-block variability. Overall, the economic performance of cowpea biofortification was strongly influenced by micronutrient management, reflecting the combined effects of grain yield response and treatment-specific production costs.

**Table 5:** Analysis of variance for the economic indicators, benefit–cost ratio (B:C) and net profit (NP), as affected by fertilization mode, zinc (Zn) rate, iron (Fe) rate, and their interactions.

Source of variation	df	Benefit–cost ratio (B:C)				Net profit (NP)			
		SS	MS	F	P	SS	MS	F	P
Block	3	0.057	0.019	545.20	<0.001	8150	2717	365.14	<0.001
Mode	2	3.083	1.541	44536.93	<0.001	29048	14524	1952.20	<0.001
Zn	3	4.527	1.509	43608.07	<0.001	1020169	340056	45707.15	<0.001
Fe	3	26.411	8.804	254392.42	<0.001	215260	71753	9644.39	<0.001
Mode × Zn	6	0.217	0.036	1045.39	<0.001	46986	7831	1052.57	<0.001
Mode × Fe	6	0.669	0.112	3223.47	<0.001	10573	1762	236.85	<0.001
Zn × Fe	9	0.396	0.044	1272.01	<0.001	74847	8316	1117.81	<0.001
Mode × Zn × Fe	18	0.057	0.003	91.27	<0.001	7491	416	55.93	<0.001
Residual	141	0.005	0.000	—	—	1049	7	—	—

**Notes:** df, degrees of freedom; SS, sum of squares; MS, mean square; F, F statistic; B:C, benefit–cost ratio; NP, net profit; Zn, zinc application rate; Fe, iron application rate; M, fertilization mode. The residual mean square (MS error) was used as the denominator for all F-tests.

### Economic response surface for net profit

Net profit response to Zn and Fe fertilization was adequately described by a second-order polynomial regression model, which explained 86.99% of the total variation in net profit (R^2^ = 0.8699). The fitted regression equation was:


$$\begin{array}{c} \text{NP}=\text{725}.\text{916}+\text{19}.\text{535Zn}-\text{0}.\text{6299Z}{\text{n}}^{\text{2}}+\text{3}.\text{609Fe}-\text{0}.\text{3295F}{\text{e}}^{\text{2}}+\text{0}.\text{1793}(\text{Zn}\times \text{Fe}) \end{array}$$ (12)

**Fig 8:**
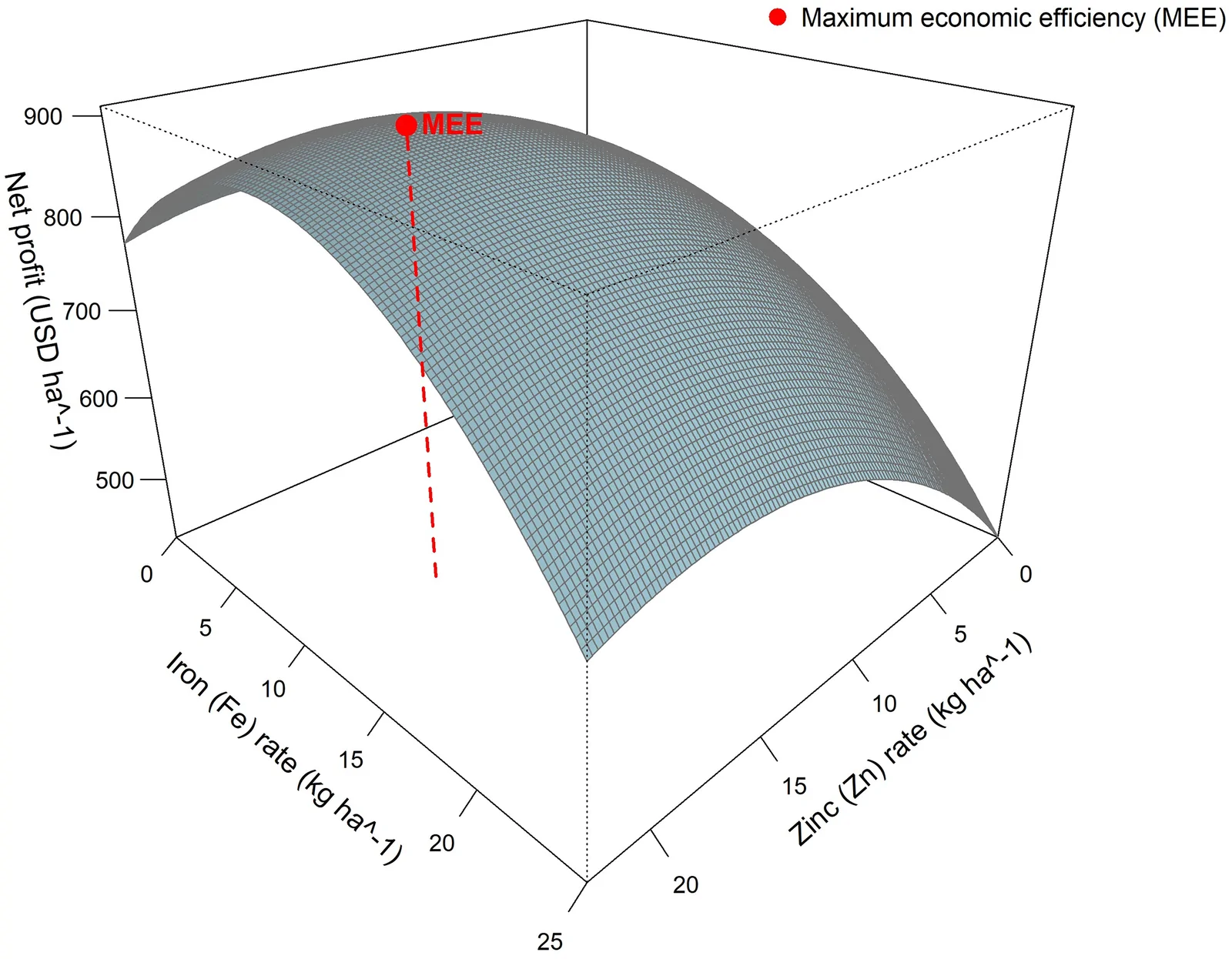
Response surface showing the effect of Zn and Fe fertilization on net profit (USD ha ⁻ ¹).

All model coefficients were statistically significant. The positive linear coefficients for Zn and Fe indicate that net profit initially increased with increasing micronutrient rates, whereas the negative quadratic terms show diminishing economic returns beyond the optimum. The positive interaction term further indicates that combined Zn and Fe application enhanced profitability up to a certain threshold. The economic response surface is shown in Fig 8.

### Maximum economic efficiency (MEE)

Optimization of the economic response surface identified the maximum economic efficiency (MEE) at 16.94 kg ha ⁻ ¹ Zn and 10.09 kg ha ⁻ ¹ Fe. At this combination, the predicted net profit reached USD 909.57 ha ⁻ ¹. This result indicates that the economic optimum occurred at lower fertilizer rates than the agronomic optimum, particularly for Fe.

**Fig 9:**
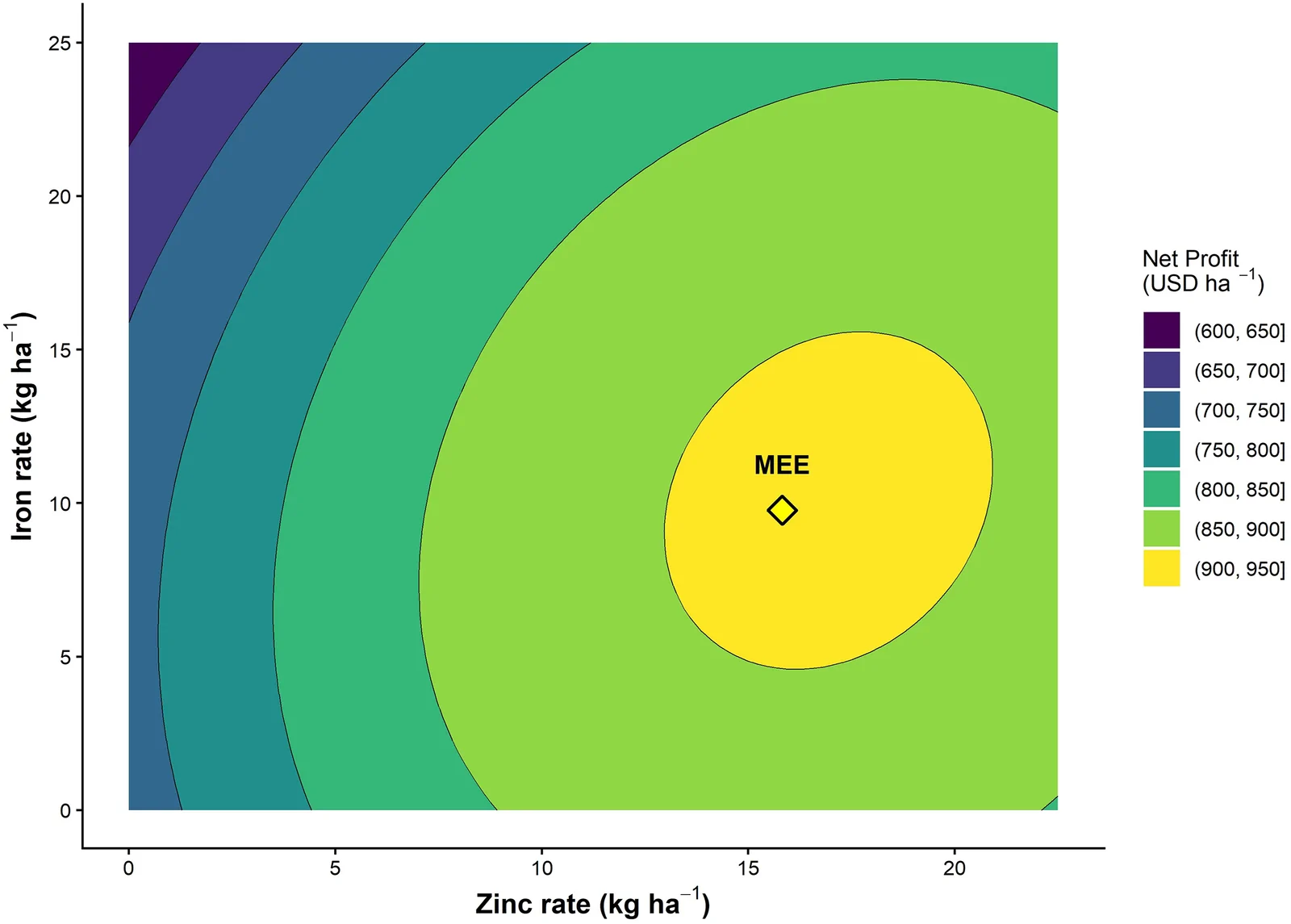
Contour plot illustrating the economic optimum (maximum economic efficiency, MEE) for Zn and Fe fertilization.

The lower economic optimum for Fe was mainly associated with the relatively high cost of Fe fertilizer, which reduced profitability at higher application rates despite continued increases in grain yield. Thus, maximum economic return was achieved before the point of maximum biological productivity was reached. The location of the economic optimum is illustrated in Fig 9. These findings were further reinforced by sensitivity and stochastic risk analyses, which confirmed the stability and robustness of the economic optimum under variable production and market conditions.

### Agronomic versus economic optimization

Comparison between agronomic and economic optimization revealed distinct micronutrient requirements for maximizing grain yield and maximizing economic return (Table 6). Maximum technical efficiency (MTE) was achieved at 18.92 kg Zn ha ⁻ ¹ and 19.17 kg Fe ha ⁻ ¹, corresponding to a predicted grain yield of 1875.97 kg ha ⁻ ¹. In contrast, maximum economic efficiency (MEE) occurred at 16.94 kg Zn ha ⁻ ¹ and 10.09 kg Fe ha ⁻ ¹, producing a predicted grain yield of 1838.21 kg ha ⁻ ¹ and a predicted net profit of USD 909.57 ha ⁻ ¹.

**Table 6:** Comparison between the maximum technical efficiency (MTE) and maximum economic efficiency (MEE) for Zn and Fe fertilization in cowpea.

Optimization criterion	Zn rate (kg ha ⁻ ¹)	Fe rate (kg ha ⁻ ¹)	Predicted grain yield (kg ha ⁻ ¹)	Net profit (USD ha ⁻ ¹)	Fertilizer cost (USD ha ⁻ ¹)
Maximum technical efficiency (MTE)	18.92	19.17	1875.97	883.14	124.46
Maximum economic efficiency (MEE)	16.94	10.09	1838.21	909.57	71.58
Change relative to MTE (%)	−10.48	−47.39	−2.01	+2.99	−42.50

**Notes:** MTE, maximum technical efficiency; MEE, maximum economic efficiency. Relative changes were calculated using MTE as the reference. Fertilizer cost includes only Zn and Fe fertilizer costs and excludes baseline production and application costs.

Relative to the agronomic optimum, the economic optimum reduced Zn and Fe application rates by 10.48% and 47.39%, respectively. This reduction resulted in only a 2.01% decrease in predicted grain yield while reducing fertilizer costs by 42.50%. Consequently, net profit increased by 2.99%, from USD 883.14 ha ⁻ ¹ at MTE to USD 909.57 ha ⁻ ¹ at MEE. These results demonstrate that maximizing economic return required substantially lower micronutrient inputs than maximizing biological productivity, particularly for Fe, highlighting the value of bioeconomic optimization for fertilizer recommendation under the conditions evaluated

### Economic risk and sensitivity analysis

To further evaluate the robustness of the proposed fertilization strategies, complementary break-even, risk, and sensitivity analyses were performed using the observed experimental dataset.

**Fig 10:**
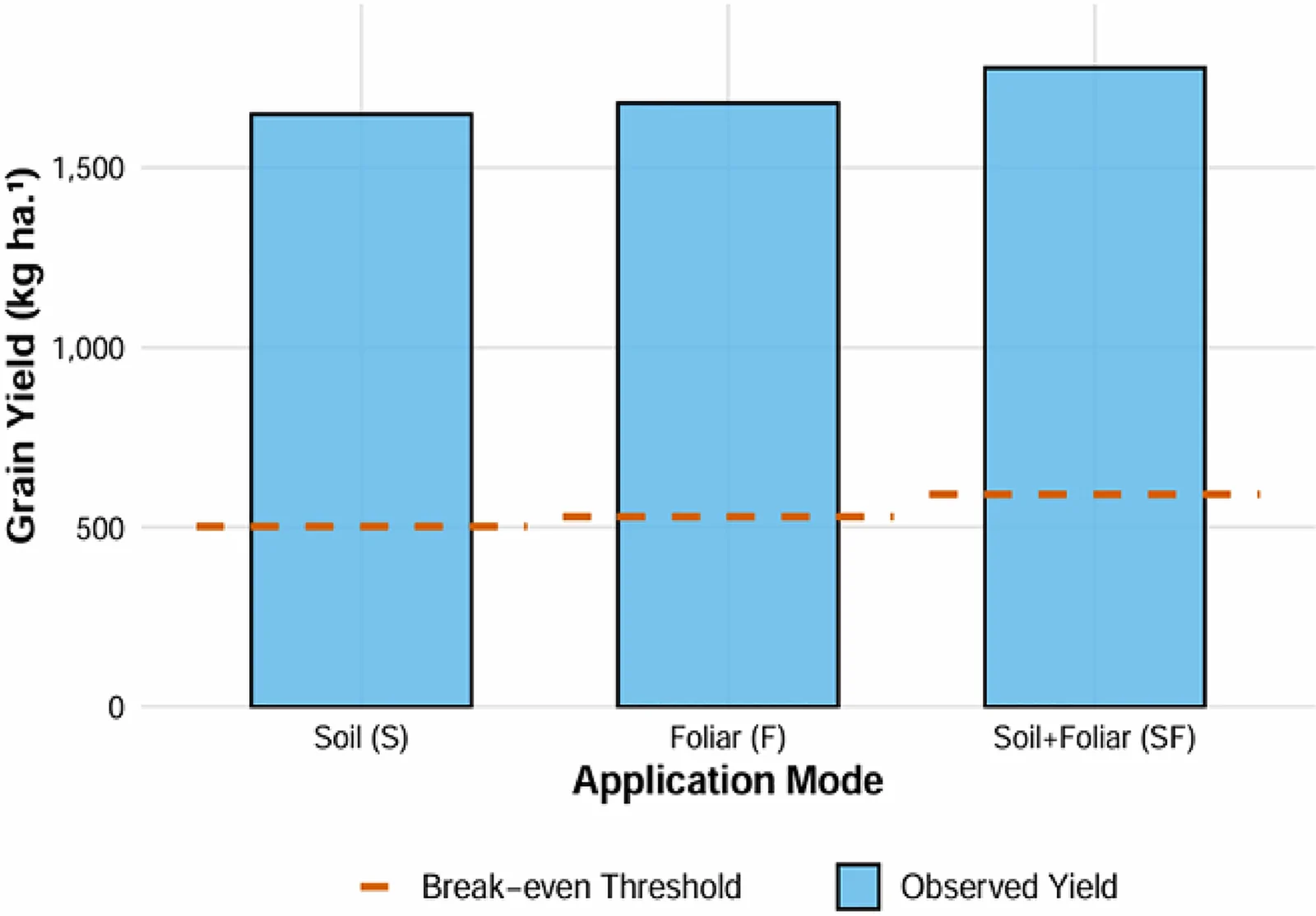
Break-even analysis showing the minimum grain yield required to offset production costs under different fertilization strategies.

Break-even analysis indicated that all treatments produced grain yields substantially above the minimum threshold required to offset production costs, confirming the economic feasibility of all fertilization strategies evaluated (Fig 10). Although the combined soil plus foliar application (SF) involved higher operational costs, it consistently generated the highest economic returns, whereas the soil-only (S) application required the lowest break-even yield to achieve profitability.

**Fig 11:**
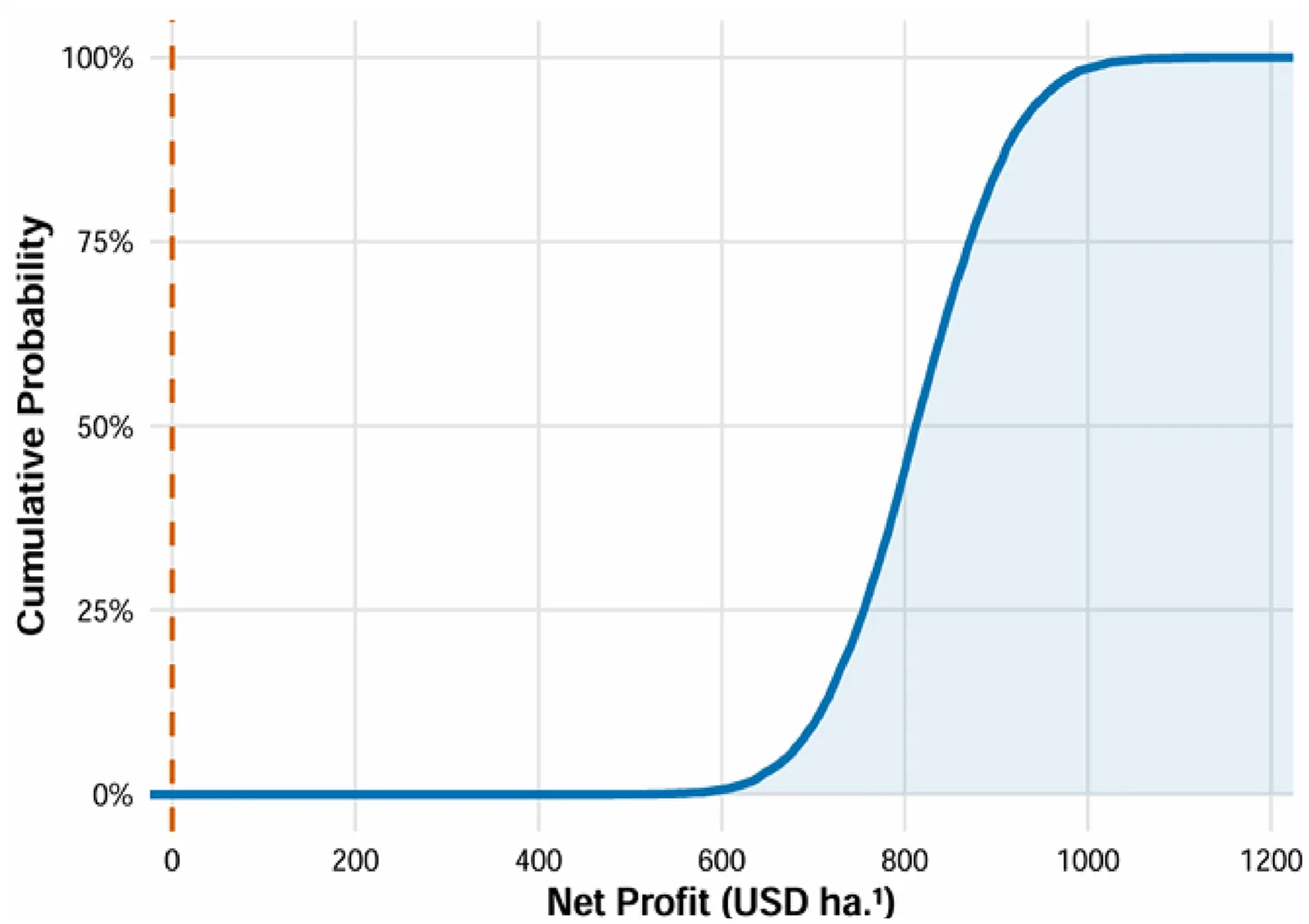
Cumulative distribution function of net profit (USD ha ⁻ ¹), showing the probability distribution of economic returns across fertilization treatments.

The empirical distribution of net profit showed a mean value of USD 812.87 ha ⁻ ¹ with a standard deviation of USD 86.03 ha ⁻ ¹, indicating relatively low variability in economic returns. All evaluated treatments generated positive net profits within the observed experimental dataset, and none of the evaluated treatment combinations resulted in a negative net profit under the agronomic and economic conditions considered in this study. This pattern is illustrated by the cumulative distribution function (Fig 11), in which the distribution of net profits remained above the break-even threshold.

**Fig 12:**
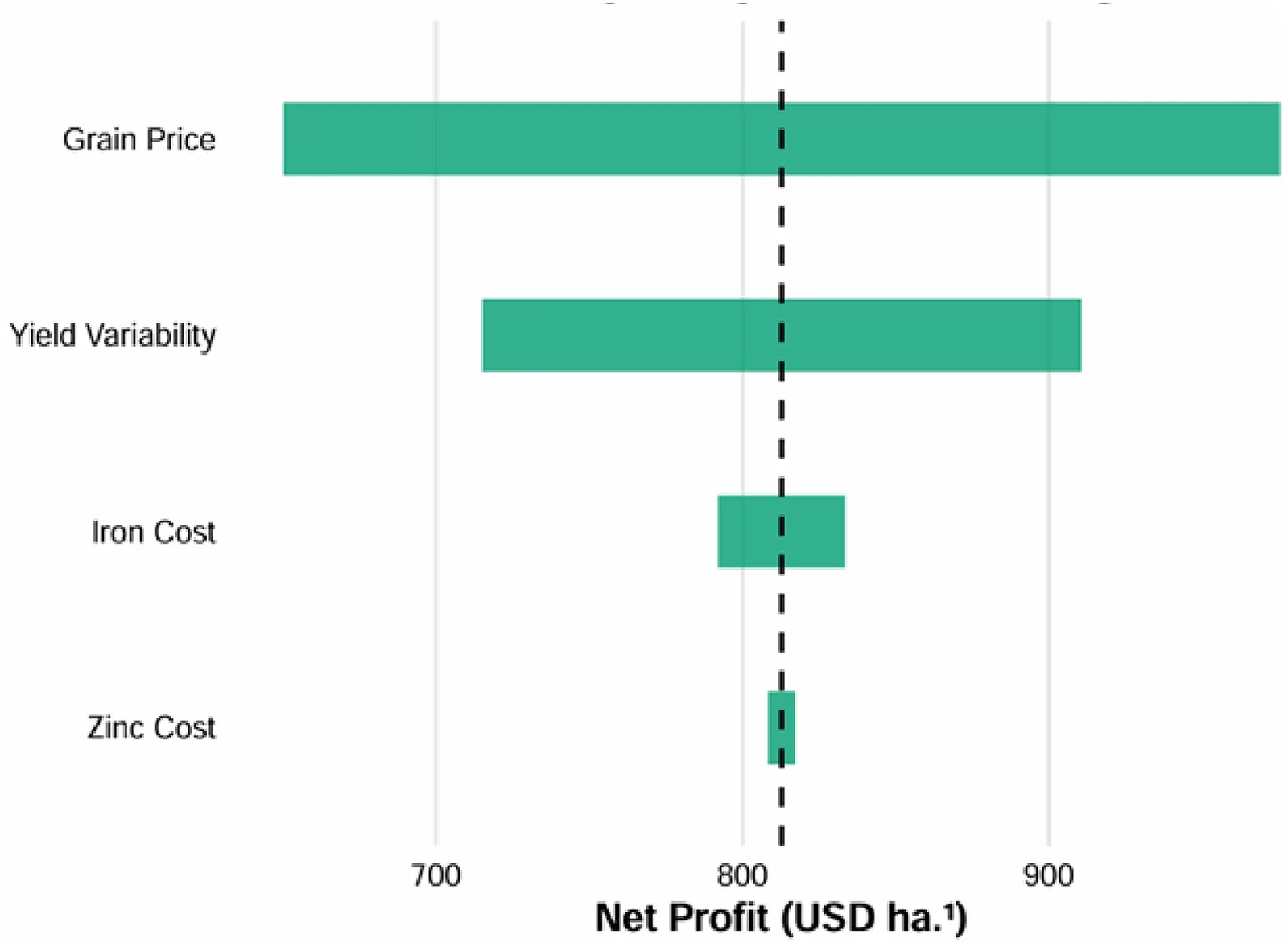
Tornado diagram showing the relative influence of ±20% changes in grain price, grain yield, zinc fertilizer cost, and iron fertilizer cost on net profit.

Sensitivity analysis was conducted using the predicted maximum economic efficiency (baseline net profit = USD 909.57 ha ⁻ ¹) as the reference scenario. Grain price, grain yield, zinc fertilizer cost, and iron fertilizer cost were independently varied by ±20% relative to their baseline values while maintaining the remaining variables constant. Thus, each variable was evaluated at 80% (low) and 120% (high) of its baseline value to quantify its influence on net profit. The tornado diagram (Fig 12) showed that grain price and grain yield were the variables exerting the greatest influence on net profit, whereas the costs of zinc and iron fertilizers had comparatively smaller effects. These results indicate that the economic performance of the proposed fertilization strategies is primarily driven by market price and productivity fluctuations, while remaining relatively insensitive to plausible variations in micronutrient fertilizer costs.

## Discussion

### Soil micronutrient limitation as a primary driver of cowpea productivity

The results clearly demonstrate that cowpea productivity in the studied environment was primarily constrained by severe zinc deficiency, as evidenced by the extremely low DTPA-extractable Zn concentration (0.1 mg kg ⁻ ¹). This value is well below the critical threshold required for optimal legume production, confirming Zn limitation as the dominant yield-restricting factor [23]. Accordingly, these findings are consistent with previous studies highlighting widespread Zn deficiency in highly weathered tropical soils of Sub-Saharan Africa [9,24]. Physiologically, Zn plays a central role in enzymatic activation, protein synthesis, and hormonal regulation, particularly auxin metabolism, which directly influences root growth and reproductive development [11,25]. Thus, the strong yield response observed most likely reflects the restoration of key metabolic functions impaired under Zn deficiency. Although soil Fe levels were relatively high, the significant Zn × Fe interaction indicates that plant performance depends on nutrient balance rather than the absolute availability of individual micronutrients. Collectively, these findings reinforce the concept that crop nutritional responses arise from integrated interactions among soil nutrient availability, nutrient acquisition, and plant physiological processes rather than from isolated nutrient effects. Accordingly, the agronomic responses observed in the present study are best interpreted as the outcome of coordinated soil–plant interactions regulating Zn and Fe availability, uptake, and utilization under the experimental conditions [26–28].

### Synergistic interaction between zinc and iron

The positive Zn × Fe interaction identified by the response surface model suggests a synergistic effect between these micronutrients under the experimental conditions. Although antagonistic interactions between Zn and Fe have been reported under excessive nutrient supply, previous studies have shown that their combined application under deficient conditions may enhance nutrient acquisition, improve physiological performance, and increase crop productivity [16,17].

The physiological mechanisms underlying this response were not directly evaluated in the present study. Nevertheless, previous research suggests that Zn may contribute to improved root development and membrane integrity, potentially enhancing Fe acquisition, whereas Fe is involved in chlorophyll synthesis and electron transport, thereby supporting photosynthetic activity [29–31].

Furthermore, interactions between Zn and Fe may influence nutrient uptake and homeostasis at the soil–plant interface [26]. Accordingly, the positive interaction observed in this study is interpreted as a plausible consequence of complementary physiological processes associated with balanced micronutrient nutrition rather than as direct evidence of specific physiological mechanisms.

Likewise, synergistic responses have been reported in cereals and grain legumes, where integrated Zn and Fe management enhanced nutrient uptake, physiological performance, grain micronutrient accumulation, and crop productivity [11,27,28].

Moreover, previous studies have shown that root-induced rhizosphere modifications, including localized pH changes, alterations in rhizosphere chemistry, and the release of root exudates, may influence Zn and Fe availability and uptake [32,33]. Although these processes were not investigated in the present study, they may also have contributed to the positive interaction observed under the experimental conditions.

### Fertilization strategy and nutrient use efficiency

The superior performance of the combined soil + foliar application highlights the importance of synchronizing nutrient supply with crop physiological demand. Soil application provides a continuous nutrient supply during the early stages of crop establishment, promoting root development and initial plant growth, whereas foliar application rapidly supplies nutrients during periods of high demand, particularly at the reproductive stage [33]. Consequently, the combination of these application methods improves the temporal synchronization between nutrient availability and crop requirements, thereby increasing nutrient recovery and agronomic efficiency. Comparable responses have been reported in studies demonstrating that integrated fertilization strategies improve nutrient-use efficiency by better synchronizing nutrient availability with crop demand throughout the growing season [34,35].

Moreover, the combined strategy may improve nutrient-use efficiency by complementing soil nutrient availability with direct foliar uptake during critical growth stages, thereby potentially reducing nutrient losses associated with soil fixation and leaching. Although these processes and nutrient uptake pathways were not directly evaluated in the present study, the present interpretation is consistent with previous reports showing that integrated micronutrient management enhances nutrient uptake, physiological performance, biomass accumulation, and grain productivity compared with individual fertilization strategies [16,36].

### Bioeconomic optimization and diminishing returns

A major contribution of the present study is the clear distinction between the agronomic and economic optima for Zn and Fe fertilization in cowpea. Whereas maximum technical efficiency (MTE) was achieved at relatively higher micronutrient application rates, maximum economic efficiency (MEE) was attained at substantially lower fertilizer inputs, particularly for Fe. This divergence demonstrates that the fertilizer combination maximizing biological productivity does not necessarily maximize economic return, especially when fertilizer costs are explicitly incorporated into the optimization process.

This pattern is consistent with the principle of diminishing marginal returns, whereby successive increases in fertilizer application produce progressively smaller increments in grain yield that eventually become insufficient to compensate for the additional production costs. Similarly, fertilizer optimization studies have shown that recommendations based exclusively on maximum biological yield frequently exceed economically optimal fertilizer rates, particularly under conditions of increasing fertilizer prices and fluctuating grain market values [18,37].

The relatively high cost of Fe fertilizer was the principal factor responsible for the divergence between the agronomic and economic optima observed in the present study. Although grain yield continued to increase with Fe application until reaching the biological optimum, the fitted response surface indicated progressively smaller yield gains as Fe rates increased. As a result, the economically optimal solution was achieved at a substantially lower Fe application rate, reducing Fe input by approximately 47% while maintaining more than 97% of the maximum predicted grain yield. Overall, these findings demonstrate that moderate reductions in fertilizer inputs can substantially improve economic performance while causing only negligible reductions in biological productivity.

From a practical perspective, the results emphasize the importance of integrating agronomic and economic criteria when developing fertilizer recommendations for smallholder production systems. Recommendations based solely on maximum yield may encourage fertilizer application rates that are agronomically effective but economically inefficient. Conversely, bioeconomic optimization provides a more robust decision-support framework by simultaneously considering crop response, fertilizer costs, and market returns, thereby maximizing farm profitability while maintaining high levels of productivity [38,39].

Nevertheless, the economic optimum identified in this study should be regarded as inherently context-specific. Variations in fertilizer prices, grain market values, labor costs, and local production conditions may alter the economically optimal combination of Zn and Fe application rates. Therefore, although the methodological framework presented here is broadly applicable, optimum fertilizer recommendations should be locally calibrated to reflect the economic conditions of different production environments [40,41].

### Robustness of the economic optimum under uncertainty

Under the agronomic, environmental, and economic conditions evaluated, all fertilization treatments generated positive net returns within the observed dataset, indicating consistently favorable economic performance across the scenarios analyzed. Collectively, these results indicate that agronomic biofortification with Zn and Fe represents a relatively low-risk investment under the conditions evaluated in this study.

Furthermore, sensitivity analysis identified grain price and grain yield as the principal determinants of economic performance, whereas variations in Zn and Fe fertilizer costs had comparatively smaller effects on net profitability. These findings suggest that market fluctuations and crop productivity exert a substantially greater influence on economic returns than moderate changes in micronutrient fertilizer prices. Likewise, recent studies have demonstrated that uncertainty in crop yield and market prices constitutes one of the major sources of variability in fertilizer recommendation strategies and farm profitability, emphasizing the importance of incorporating economic risk into fertilizer management decisions [42].

From an applied perspective, the combined soil + foliar application of approximately 16–17 kg Zn ha ⁻ ¹ and 10 kg Fe ha ⁻ ¹ provided the most favorable balance between grain productivity and economic return under the conditions of the present study. Nevertheless, because the economically optimal fertilizer combination depends on prevailing fertilizer prices, grain market values, labor costs, and local production conditions, these recommendations should be regarded as site-specific rather than universally applicable. Accordingly, fertilizer recommendation strategies should be periodically refined to reflect local agronomic and economic conditions while also considering farmers’ access to agricultural inputs, extension services, and institutional support to facilitate adoption. Such an integrated approach is essential for improving farm profitability, reducing production risks, and promoting the long-term sustainability of nutrient management in smallholder farming systems [43].

The significant block effects observed for both grain yield and the economic indicators indicate that the randomized complete block design successfully accounted for spatial variability across the experimental area. Although some environmental heterogeneity was present among blocks, treatment effects remained substantially greater than block-to-block variation, as demonstrated by the markedly higher F-values associated with fertilization mode and micronutrient application rates

### Implications for sustainable intensification and policy

Beyond the agronomic and economic benefits demonstrated at the farm level, the present findings have broader implications for sustainable agricultural intensification and food and nutrition security in Sub-Saharan Africa. Agronomic biofortification with Zn and Fe represents a practical strategy to simultaneously increase crop productivity and improve the nutritional quality of staple foods, thereby contributing to efforts aimed at reducing micronutrient deficiencies while strengthening the resilience of smallholder farming systems [8,44].

Importantly, the present study demonstrates that economically optimized micronutrient fertilization can substantially reduce fertilizer inputs without significantly compromising grain yield. Under the conditions evaluated, the economically optimal fertilization strategy required considerably lower Fe application than the agronomic optimum while maintaining more than 97% of the maximum predicted yield. These findings underscore the value of integrating agronomic and economic criteria to improve nutrient-use efficiency, increase farm profitability, and promote more sustainable fertilizer management [39,45].

From a policy perspective, the results indicate that fertilizer recommendation programs should move beyond yield maximization and incorporate economic optimization to better reflect farmers’ production realities. Decision-support approaches integrating crop response, fertilizer costs, and market prices can provide more efficient and economically viable recommendations, particularly for resource-constrained smallholder farmers [46,47].

Equally important, the successful implementation of agronomic biofortification at larger scales will depend not only on scientifically sound fertilizer recommendations but also on farmers’ access to micronutrient fertilizers, effective agricultural extension services, reliable input supply systems, and supportive public policies. Therefore, strengthening these institutional and technological components will be essential to facilitate the adoption of integrated nutrient management practices and maximize their contribution to sustainable agricultural intensification, improved livelihoods, and food and nutrition security particularly in Sub-Saharan Africa [8,43].

## Conclusion

This study demonstrates that integrating agronomic and economic optimization provides a more efficient framework for defining Zn and Fe fertilization strategies than approaches based solely on maximum biological yield. Under the tropical field conditions of Mozambique evaluated in this study, the economically optimal fertilization strategy required lower micronutrient inputs than the agronomic optimum, particularly for Fe, while maintaining more than 97% of the maximum predicted grain yield and improving economic return.

The combined soil + foliar fertilization strategy consistently produced the greatest grain yield and agronomic efficiency, highlighting the importance of synchronizing micronutrient supply with crop demand. These findings indicate that bioeconomic optimization can substantially improve fertilizer recommendations by reducing unnecessary micronutrient inputs while maintaining high productivity and profitability under the conditions evaluated.

Because this study was conducted at a single experimental site during one growing season, the proposed fertilizer recommendations should be interpreted as specific to the environmental, agronomic, and economic conditions investigated. Additional multi-location and multi-season studies across contrasting soil types, climatic conditions, and production environments are needed to validate the robustness of the proposed optimum fertilization strategy. Future research should integrate agronomic biofortification with genotype selection, grain micronutrient accumulation, and multi-environment validation to further improve the efficiency, robustness, and scalability of Zn and Fe fertilization strategies for cowpea production under tropical conditions.

## Supporting information

S1 Dataset Experimental dataset used for the statistical analyses, including block, fertilization mode, zinc (Zn) and iron (Fe) application rates, and grain yield. (XLSX)
