# Supplementary material for: Bioeconomic optimization of zinc and iron agronomic biofortification in cowpea (Vigna unguiculata [L.] Walp.) in Mozambique under tropical field conditions
Source: PLoS One. 2026 Sep 3;21(9):e0357473. doi: 10.1371/journal.pone.0357473 (PMC13541262; doi:10.1371/journal.pone.0357473)
Supplement: Supplementary file 1 [file pone.0357473.s003.docx]

**Response to the Academic Editor and Reviewers**

**Manuscript ID: PONE-D-26-24166**

Title:
**Bioeconomic optimization of zinc and iron agronomic biofortification in cowpea (Vigna unguiculata [L.] Walp.) under tropical field conditions in Mozambique**

**Dear Academic Editor and Reviewers,**

We sincerely thank the Academic Editor and both reviewers for their careful evaluation of our manuscript and for their constructive comments and insightful suggestions. We greatly appreciate the time and effort invested in reviewing our work, as well as the recognition of its scientific relevance, technical soundness, experimental design, and practical importance.

In response to the comments received, we have thoroughly revised the manuscript. The major revisions include clarification of the experimental design and statistical methodology, complete reanalysis of the factorial analysis of variance (ANOVA), revision of the bioeconomic optimization framework, improvement of the soil and climatic characterization, moderation of physiological interpretations, acknowledgment of the limitations associated with a single-site and single-season experiment, incorporation of recent and directly relevant literature, revision of the figures and tables, and comprehensive editing of the English language and manuscript formatting.

We have carefully addressed every comment raised by the reviewers. Below, each reviewer comment is reproduced in bold, followed by our detailed response and a summary of the corresponding revisions made to the manuscript. All modifications are highlighted in the revised manuscript submitted with tracked changes.

We hope that the revisions have satisfactorily addressed all concerns and have substantially improved the quality, clarity, and scientific rigor of the manuscript.

Sincerely,

**Tancredo José Carlos**
On behalf of all authors

**Responses to Journal Requirements**

**Journal Requirement 1**

**Comment:**
Please ensure that your manuscript meets PLOS ONE’s style requirements, including those for file naming.

**Response:**
We sincerely thank the journal for this reminder. The revised manuscript has been carefully reviewed and reformatted to comply with the PLOS ONE style requirements. Terminology, units, abbreviations, table and figure presentation, reference formatting, and file naming have been standardized throughout the submission.

**Changes made:**

The manuscript formatting was revised according to the PLOS ONE guidelines.

Units, abbreviations, tables, figures, and references were standardized.

The revised files will be uploaded using the required designations: Manuscript, Revised Manuscript with Track Changes, and Response to Reviewers.

**Journal Requirement 2**

**Comment:**
The grant information provided in the “Funding Information” and “Financial Disclosure” sections does not match. Please provide the correct grant numbers.

**Response:**
We sincerely thank the journal for identifying this inconsistency. The funding information has been reviewed and corrected to ensure that the grant number and funding organization are reported consistently in the manuscript, cover letter, and online submission system.

Revised funding statement:

This study was supported by the Regional Scholarship and Innovation Fund (RSIF), a program of the Partnership for Skills in Applied Sciences, Engineering and Technology (PASET), under grant number B8501N10019 awarded to Tancredo José Carlos.

**Changes made:**
The Funding Information and Financial Disclosure statements were harmonized to report the same funding organization and grant number.

**Journal Requirement 3**

**Comment:**
Please state what role the funders took in the study. If the funders had no role, please state: “The funders had no role in study design, data collection and analysis, decision to publish, or preparation of the manuscript.”

**Response:**
We confirm that the funders had no role in the scientific design, execution, analysis, interpretation, or publication decisions associated with this study.

Revised Role of Funder statement:

The funders had no role in study design, data collection and analysis, decision to publish, or preparation of the manuscript.

**Changes made:**
This statement has been included in the revised manuscript and will also be provided in the revised online submission information to ensure consistency between the manuscript and the submission system.

**Journal Requirement 4**

**Comment:**
All data underlying the findings must be freely available through a public repository, within the manuscript, or as Supporting Information.

**Response:**
We sincerely thank the journal for this clarification. The data underlying the findings of this study will be made freely available as Supporting Information accompanying the revised manuscript. The dataset includes the experimental factors, block structure, fertilization modes, Zn and Fe application rates, grain yield, and the variables used in the agronomic and economic analyses.

Revised Data Availability statement:

All data underlying the findings reported in this study are provided in the Supporting Information files accompanying the manuscript.

**Changes made:**

The underlying experimental dataset will be uploaded as Supporting Information.

The Data Availability statement has been revised accordingly.

**Journal Requirement 5**

**Comment:**
Please upload a new copy of Figure 1 because the details are not clear.

**Response:**
We sincerely thank the journal for this comment. Figure 1 has been replaced with a higher-resolution version. Font sizes, labels, geographical boundaries, symbols, and annotations were enlarged and standardized to improve readability.

**Changes made:**
Figure 1 was regenerated and replaced with a clearer, higher-resolution version that complies with the PLOS ONE figure preparation requirements.

**Journal Requirement 6**

**Comment:**
Figure 1 may contain copyrighted map images. Please provide permission or replace the figure with a map that complies with the CC BY 4.0 license.

**Response:**
We sincerely thank the journal for drawing our attention to this issue. The original map was replaced with a newly generated figure based exclusively on open-access or public-domain spatial data. No Google Maps, Google Earth, proprietary satellite imagery, or other copyrighted map material was used in the revised figure.

**Changes made:**

Figure 1 was reconstructed using spatial data compatible with open-access publication under the CC BY 4.0 license.

The figure caption was revised to identify the spatial data source.

The previous map containing potentially copyrighted material was removed.

Figure caption source statement:

Administrative boundaries and base-map data were obtained from open-access spatial datasets and the figure was created by the authors

**Journal Requirement 7**

**Comment:**
Table 2 is not referred to in the manuscript text. Please ensure that Table 2 is cited in the text.

**Response:**
We sincerely thank the journal for identifying this omission. In the revised manuscript, the factorial analysis of variance table, originally presented as Table 2, has been renumbered as **Table 3** following the inclusion of a new table describing the climatic conditions during the experimental season. The Results section has been revised to refer explicitly to **Table 3** when presenting the factorial analysis of variance and the significance of the main effects and interaction terms.

**Changes made:**
A direct citation to **Table 3** was added to the Results section, ensuring that the factorial analysis of variance table is properly linked to the corresponding statistical description in the text.

**Reviewer #1**

We sincerely thank Reviewer #1 for the thorough evaluation of our manuscript and for the constructive comments and insightful suggestions. We greatly appreciate the reviewer's careful assessment and the positive feedback regarding the scientific quality of our study. The comments have substantially improved the clarity, rigor, and interpretation of the manuscript. We have carefully considered each recommendation and revised the manuscript accordingly. Our point-by-point responses are presented below.

**Comment 1**

Reviewer comment

Please report the WRB or USDA soil classification of the experimental site.

**Response**

We sincerely thank the reviewer for this valuable comment. Unfortunately, a site-specific WRB or USDA soil classification was not available for the experimental field. Therefore, we chose not to assign a formal soil classification without direct pedological characterization, as doing so could result in an inaccurate or unsupported classification. Instead, the soil was characterized based on its measured physical and chemical properties obtained before crop establishment, including texture, pH, organic carbon, available phosphorus, exchangeable potassium, sulfur, DTPA-extractable zinc and iron, and total nitrogen. To provide regional pedological context, we also incorporated information from the National Soil Map of Mozambique, which indicates that the study area is predominantly characterized by Arenosols, Ferralsols, Lixisols, and Acrisols. We believe that this approach provides a scientifically sound description of the experimental soil while avoiding unsupported taxonomic classification.

**Changes made in the manuscript**

The description of the experimental soil in the Materials and Methods section was revised to clarify that the soil was characterized based on its measured physical and chemical properties rather than assigning an unsupported WRB or USDA classification. In addition, regional soil information from the National Soil Map of Mozambique was included to provide pedological context.

**Comment 2**

Reviewer comment

Please report whether the experimental season was climatically representative of the long-term average for the region.

**Response**

We sincerely thank the reviewer for this valuable comment. To address this suggestion, we compared the climatic conditions recorded during the 2025 growing season with the long-term climatic normals obtained from the Quelimane Meteorological Station, the closest meteorological station to the experimental site. To facilitate this comparison, we included a new table in the revised manuscript summarizing the observed and long-term climatic conditions. Mean monthly temperatures during the experimental period were very similar to the long-term averages, with only minor differences, while rainfall exhibited some monthly variability but maintained the characteristic seasonal distribution of the region. Overall, this comparison indicates that the experiment was conducted under climatic conditions that were broadly representative of the regional production environment.

**Changes made in the manuscript**

A new table comparing the climatic conditions observed during the 2025 growing season with the long-term climatic normals for the Quelimane Meteorological Station was added to the manuscript. The Materials and Methods and Results sections were revised accordingly to demonstrate that the experimental season was broadly representative of the climatic conditions of the study region.

**Comment 3**

Reviewer comment

Please clarify the statistical model used in the analysis and specify whether block was treated as a fixed or random effect.

**Response**

We sincerely thank the reviewer for this important comment. We carefully revised the Statistical Analysis section to provide a clearer and more accurate description of the statistical model used in this study. The treatment effects were evaluated using a factorial analysis of variance (ANOVA) for a randomized complete block design (RCBD) arranged in a 3 × 4 × 4 factorial scheme, including the effects of block, fertilization mode, Zn rate, Fe rate, and their interactions. Because a mixed-effects model was not fitted, we removed the previous statement indicating that block was treated as a random effect. The statistical model is now described in a manner that accurately reflects the analytical approach adopted in the study.

**Changes made in the manuscript**

The Statistical Analysis section was revised to provide a clear description of the factorial ANOVA model used in the study. The previous statement indicating that block was treated as a random effect was removed, and the statistical model was rewritten to accurately describe the effects included in the analysis and the experimental design

**Comment 4**

Reviewer comment

P8 L200–208: The manuscript reports significant Zn × Fe interactions. Please provide the corresponding interaction P-values within the text or refer readers directly to the ANOVA table.

**Response**

We sincerely thank the reviewer for this helpful suggestion. To improve the transparency and readability of the statistical results, we revised the Results section to explicitly report the corresponding P-values for the interaction terms discussed in the text. In addition, direct references to Table 3 were included wherever the ANOVA results are described, allowing readers to easily relate the narrative interpretation to the complete statistical analysis.

**Changes made in the manuscript**

The Results section was revised to include the corresponding P-values for the significant interaction terms discussed in the text. Direct references to Table 3 were also added to improve consistency between the narrative description and the ANOVA results.

**Comment 5**

Reviewer comment

P9 L218–226: Consider reporting confidence intervals for the predicted optimum nutrient rates, which would provide an indication of the robustness of the optimization model.

Response

We sincerely thank the reviewer for this valuable suggestion. We agree that confidence intervals for the estimated optimum nutrient rates could provide additional information regarding the uncertainty associated with the optimization results. However, estimating confidence intervals for the stationary point of a second-order response surface model requires specialized statistical procedures, such as bootstrap resampling or delta-method approaches, which were beyond the scope of the present study. To address the reviewer's concern, we clarified that the estimated optimum occurred within the experimental domain and strengthened the evaluation of model robustness by emphasizing the goodness-of-fit statistics, coefficient of determination (R²), and residual diagnostics used to assess model adequacy.

**Changes made in the manuscript**

The Statistical Analysis and Results sections were revised to clarify that the estimated optimum was located within the experimental domain. Additional information regarding model adequacy, including the coefficient of determination (R²), residual diagnostics, and model adequacy assessment, was incorporated to provide greater confidence in the robustness of the response surface model.

**Comment 6**

Reviewer comment

P10 L236–245: The response surfaces are informative. Adding contour values or predicted yield labels would facilitate interpretation of the biological optimum.

Response

We sincerely thank the reviewer for this valuable suggestion. We agree that adding contour labels could further facilitate the interpretation of the response surface. However, the contour plot already clearly identifies the optimum region corresponding to the maximum predicted grain yield. Rather than adding numerical labels that could reduce figure readability, we revised the figure legend to explicitly state that the contour plot represents the predicted grain yield and highlights the region corresponding to the Maximum Technical Efficiency (MTE). We believe this revision improves figure interpretation while preserving its visual clarity.

**Changes made in the manuscript**

The legend of Fig 5 was revised to clarify that the contour plot represents predicted cowpea grain yield and to explicitly identify the estimated Maximum Technical Efficiency (MTE), including the corresponding Zn and Fe application rates and predicted grain yield. This revision improves interpretation of the biological optimum while preserving the visual clarity of the figure.

**Comment 7**

Reviewer comment

P11 L252–260: The grain Zn and Fe concentrations increased substantially. Please discuss whether dilution effects associated with higher grain yield were considered during data interpretation.

**Response**

We sincerely thank the reviewer for this valuable comment. We agree that dilution effects are an important consideration when interpreting changes in grain micronutrient concentrations. However, in the revised version of the manuscript, grain Zn and Fe concentrations are no longer presented because the scope of this study focuses exclusively on grain yield, agronomic efficiency, and the bioeconomic optimization of Zn and Fe fertilization. Consequently, the interpretation of dilution effects associated with grain micronutrient concentrations is beyond the scope of the present manuscript. We acknowledge the importance of this topic and agree that it should be considered in studies specifically designed to evaluate grain mineral concentration and nutritional quality.

**Changes made in the manuscript**

The manuscript was revised to clearly define its scope as the evaluation of grain yield, agronomic efficiency, and bioeconomic optimization of Zn and Fe fertilization. Since grain micronutrient concentrations are no longer presented, discussion of dilution effects was removed, as it is not applicable to the objectives of the revised manuscript.

**Comment 8**

Reviewer comment

P13 L285–294: Please clarify whether nutrient accumulation was calculated on a concentration basis or total nutrient content per unit area, since these parameters provide different physiological information.

**Response**

We sincerely thank the reviewer for this valuable comment. We agree that nutrient concentration and total nutrient accumulation provide different physiological information and should be clearly distinguished when both are evaluated. However, in the revised version of the manuscript, nutrient accumulation was neither calculated nor reported. The present study focuses exclusively on grain yield, agronomic efficiency, and the bioeconomic optimization of Zn and Fe fertilization. Therefore, the distinction between concentration-based and area-based nutrient accumulation is not applicable to the scope of the revised manuscript.

**Changes made in the manuscript**

The objectives and scope of the manuscript were revised to clearly indicate that the study focuses on grain yield, agronomic efficiency, and bioeconomic optimization of Zn and Fe fertilization. Since nutrient accumulation was not evaluated, all references to nutrient accumulation were removed to avoid ambiguity and maintain consistency throughout the manuscript.

**Comment 9**

Reviewer comment

P14 L300–308: The economic analysis is one of the strengths of the manuscript. Nevertheless, indicate the fertilizer prices and grain market prices used, together with their reference period.

**Response**

We sincerely thank the reviewer for this positive and constructive comment. We appreciate the recognition of the economic analysis as a strength of our study. In response to the reviewer's suggestion, we revised the Economic Analysis section to explicitly report the fertilizer prices, grain market price, and their corresponding reference period. Specifically, fertilizer prices were obtained from commercial suppliers in Mozambique during the 2025 cropping season, while the cowpea grain price (USD 0.70 kg⁻¹) represents the average farm-gate price observed during the 2025 harvest period. These additions improve the transparency and reproducibility of the economic analysis.

**Changes made in the manuscript**

The Economic Analysis section was revised to explicitly report the fertilizer prices, the cowpea grain price, and the corresponding reference period used in all economic calculations. This information was added to improve the transparency and reproducibility of the bioeconomic analysis.

**Comment 10**

Reviewer comment

P15 L309–317: It would be useful to mention whether sensitivity analyses were conducted for different fertilizer price scenarios.

**Response**

We sincerely thank the reviewer for this valuable suggestion. To improve the transparency of the economic analysis, we revised the manuscript to provide a more detailed description of the sensitivity analysis. Rather than evaluating multiple fertilizer price scenarios simultaneously, the analysis examined the effects of independent ±20% changes in the principal economic variables, including grain price, grain yield, zinc fertilizer cost, and iron fertilizer cost, while all remaining variables were held constant. This approach allowed us to quantify the relative influence of each variable on net profit and to identify the factors with the greatest impact on the economic performance of the fertilization strategy.

**Changes made in the manuscript**

The Economic Analysis section was expanded to clarify the sensitivity analysis methodology. The revised text now specifies that grain price, grain yield, zinc fertilizer cost, and iron fertilizer cost were independently varied by ±20%, while all other variables remained constant. This additional information improves the transparency and reproducibility of the economic analysis.

**Comment 11**

Reviewer comment

P20 L313–316: The Discussion appropriately emphasizes that plant performance depends on nutrient balance rather than the absolute availability of Zn or Fe. This section could be strengthened by highlighting that plant nutritional responses arise from integrated interactions among nutrient acquisition, soil processes, and plant physiology rather than from isolated nutrient effects.

**Response**

We sincerely thank the reviewer for this valuable and insightful suggestion. We fully agree that plant nutritional responses should be interpreted as the result of integrated interactions among soil nutrient availability, nutrient acquisition, and plant physiological processes rather than as isolated effects of individual nutrients. Accordingly, we expanded the Discussion to emphasize that the agronomic responses observed in this study are best interpreted as the outcome of coordinated soil–plant interactions regulating Zn and Fe availability, uptake, and utilization under the experimental conditions. To strengthen this interpretation, we also incorporated additional literature supporting the role of integrated soil–plant processes in determining crop nutritional responses.

**Changes made in the manuscript**

The Discussion section was revised to emphasize that plant nutritional performance results from integrated interactions among soil nutrient availability, nutrient acquisition, and plant physiological processes, rather than from the isolated availability of individual micronutrients. Additional references were incorporated to support this interpretation and provide a stronger physiological context for the observed responses.

**Comment 12**

Reviewer comment

P21, Lines 318–325: The discussion of Zn × Fe synergism is appropriate. However, the physiological mechanisms should be presented as plausible explanations because root architecture, transporter activity, and gene expression were not evaluated.

**Response**

We sincerely thank the reviewer for this valuable and insightful comment. We fully agree that the physiological mechanisms underlying the observed Zn × Fe interaction should not be presented as direct evidence, since root architecture, nutrient transporter activity, gene expression, and other physiological processes were not evaluated in the present study. Accordingly, we carefully revised the Discussion to present these mechanisms as plausible interpretations supported by previous literature rather than as findings directly demonstrated by our experiment. The revised text explicitly acknowledges the limitations of the present study while providing an appropriate physiological context for interpreting the observed synergistic interaction between Zn and Fe.

**Changes made in the manuscript**

The Discussion section was revised to clarify that the proposed physiological mechanisms are presented as plausible explanations supported by previous studies and not as direct evidence from the present experiment. The text now explicitly acknowledges that root architecture, transporter activity, gene expression, and related physiological processes were not evaluated under the experimental conditions.

**Comment 13**

Reviewer comment

P21, Lines 322–326: The statement that Zn improves Fe acquisition through enhanced root development should be interpreted cautiously, as this mechanism was not directly evaluated in the present study.

**Response**

We sincerely thank the reviewer for this important observation. We fully agree that this physiological mechanism should not be presented as direct evidence from the present study. Accordingly, we revised the Discussion to avoid causal statements and instead describe the potential role of Zn in enhancing Fe acquisition through improved root development as a plausible interpretation supported by previous studies. The revised text explicitly states that this mechanism was not directly investigated under the experimental conditions and is presented solely to provide a physiological context for interpreting the observed Zn × Fe interaction.

**Changes made in the manuscript**

The Discussion section was revised to moderate the interpretation of the proposed mechanism. The statement was rewritten to clarify that enhanced Fe acquisition through Zn-induced root development is a plausible explanation supported by previous literature rather than a process directly demonstrated by the present experiment.

**Comment 14**

Reviewer comment

P21, Lines 326–330: The discussion would benefit from mentioning that root-induced rhizosphere modifications, such as changes in pH and the release of root exudates, may also influence Zn and Fe availability.

**Response**

We sincerely thank the reviewer for this valuable suggestion. We agree that rhizosphere processes play an important role in regulating micronutrient availability and uptake. Accordingly, we expanded the Discussion to acknowledge that root-induced modifications of the rhizosphere, including localized changes in soil pH and the release of root exudates, may influence the availability and acquisition of Zn and Fe. We also clarified that these processes were not directly investigated in the present study and are discussed only as plausible mechanisms supported by previous literature that may help explain the observed responses.

**Changes made in the manuscript**

The Discussion section was expanded to include the potential contribution of rhizosphere processes, such as localized pH changes and root exudation, to Zn and Fe availability and uptake. The revised text explicitly states that these mechanisms were not evaluated in the present study and are presented as possible physiological explanations supported by the literature.

**Comment 15**

Reviewer comment

P22, Lines 334–338: The discussion correctly highlights the advantages of the combined soil + foliar application. However, it would be useful to explain why this strategy improved agronomic efficiency compared with single application methods.

**Response**

We sincerely thank the reviewer for this valuable suggestion. We agree that a clearer explanation of the superior performance of the combined fertilization strategy strengthens the interpretation of our results. Accordingly, we expanded the Discussion to explain that soil application provides a sustained supply of nutrients during the early stages of crop establishment, supporting root development and initial plant growth, whereas foliar application rapidly supplements nutrient demand during the reproductive stage, when nutrient requirements are greatest. The combination of these application methods improves the temporal synchronization between nutrient availability and crop demand, thereby enhancing agronomic efficiency. We also clarified that these mechanisms are presented as plausible interpretations supported by previous studies rather than as direct observations from the present experiment.

**Changes made in the manuscript**

The Discussion section was expanded to explain the physiological and agronomic basis for the superior performance of the combined soil + foliar application strategy. The revised text emphasizes the complementary roles of soil and foliar fertilization in synchronizing nutrient supply with crop demand and explicitly states that these mechanisms are proposed as plausible explanations supported by the literature rather than direct evidence from the present study.

**Comment 16**

Reviewer comment

P22, Lines 340–343: The statement that the combined strategy reduces nutrient losses through lower leaching and fixation should be interpreted more cautiously because these processes were not directly measured.

**Response**

We sincerely thank the reviewer for this important observation. We fully agree that reductions in nutrient losses through lower leaching and soil fixation cannot be presented as direct evidence because these processes were not quantified under the experimental conditions. Accordingly, we revised the Discussion to present these mechanisms as plausible explanations supported by previous literature rather than as experimentally demonstrated outcomes. The revised text explicitly acknowledges that nutrient losses through leaching and soil fixation were not measured in this study and that their potential contribution is discussed only as a possible explanation for the improved agronomic performance observed under the combined soil + foliar fertilization strategy.

**Changes made in the manuscript**

The Discussion section was revised to moderate the interpretation of nutrient loss mechanisms. The text now states that reduced leaching and soil fixation are plausible explanations supported by previous studies, while explicitly acknowledging that these processes were not directly evaluated or quantified in the present experiment.

**Comment 17**

Reviewer comment

P23, Lines 356–361: The discussion of the divergence between agronomic and economic optima is well developed. However, it would be helpful to emphasize that the economic optimum is context-dependent and may change with fertilizer prices, grain prices, or production systems.

**Response**

We sincerely thank the reviewer for this valuable and constructive suggestion. We fully agree that the economic optimum identified in this study should be interpreted within the specific agronomic and economic conditions under which the experiment was conducted. Accordingly, we expanded the Discussion to emphasize that the Maximum Economic Efficiency (MEE) is context-dependent and may vary according to fertilizer prices, grain market prices, production costs, and management systems. We also highlight that changes in these factors may alter the economically optimal fertilizer rates, reinforcing the importance of developing locally adapted bioeconomic recommendations rather than applying a single recommendation across different production environments.

**Changes made in the manuscript**

The Discussion section was revised to clarify that the economic optimum identified in this study is specific to the production and economic conditions evaluated. The revised text now emphasizes that variations in fertilizer prices, grain market prices, production costs, and farming systems may result in different economically optimal fertilizer rates, highlighting the importance of locally adapted recommendations.

**Comment 18**

Reviewer comment

P23, Lines 362–366: The statement suggesting a 100% probability of economic success should be interpreted more cautiously, as economic performance depends on factors beyond the scope of the present study.

**Response**

We sincerely thank the reviewer for this important observation. We fully agree that economic performance is influenced by several factors beyond those evaluated in the present study, including market fluctuations, seasonal climatic variability, management practices, and production risks. Accordingly, we revised the manuscript to avoid any implication of guaranteed economic success. The revised text now clarifies that the estimated Maximum Economic Efficiency (MEE) represents the economically optimal fertilization strategy under the agronomic, environmental, and economic conditions evaluated in this study and should not be interpreted as universally applicable or risk-free.

**Changes made in the manuscript**

The Discussion section was revised to moderate the interpretation of the economic optimization results. Statements implying guaranteed economic success were removed and replaced with wording emphasizing that the estimated MEE is specific to the conditions evaluated and may vary under different production, environmental, and economic scenarios.

**Comment 19**

Reviewer comment

P24, Lines 367–372: The practical implications could be strengthened by discussing fertilizer availability, extension services, and adoption by smallholder farmers.

**Response**

We sincerely thank the reviewer for this valuable suggestion. We agree that the practical application of agronomic biofortification depends not only on biological and economic performance but also on the availability of agricultural inputs and the capacity of farmers to adopt improved nutrient management practices. Accordingly, we expanded the Discussion to emphasize that the successful implementation of the proposed fertilization strategy will depend on factors such as fertilizer availability, affordability, access to extension services, and the dissemination of locally adapted nutrient management recommendations. We also highlight that these considerations are particularly relevant for smallholder farming systems in Sub-Saharan Africa, where access to agricultural inputs and technical support remains limited.

**Changes made in the manuscript**

The Discussion section was expanded to include the practical implications of implementing agronomic biofortification under smallholder farming conditions. The revised text now discusses the importance of fertilizer availability, affordability, agricultural extension services, and locally adapted recommendations to facilitate the adoption of bioeconomic nutrient management strategies.

**Comment 20**

Reviewer comment

P24, Lines 373–378: The conclusions and recommendations should be moderated because the study was conducted at a single site during one growing season.

Response

We sincerely thank the reviewer for this important and constructive comment. We fully agree that the conclusions should reflect the scope of the experimental evidence. Accordingly, we revised both the Discussion and Conclusion sections to avoid overly broad generalizations. The revised manuscript now explicitly states that the findings are based on a single-site, single-season field experiment conducted under the environmental and management conditions of the study area. We also emphasize that, although the results provide valuable evidence for agronomic and bioeconomic optimization of Zn and Fe fertilization in cowpea, further multi-location and multi-season studies are needed to validate the recommendations across different agroecological conditions.

**Changes made in the manuscript**

The Discussion and Conclusion sections were revised to explicitly acknowledge the limitations associated with a single-site and single-season experiment. The conclusions were moderated to avoid broad generalizations, and a recommendation for future multi-location and multi-season validation studies was added to define the appropriate scope of applicability of the findings.

**Comment 21**

Reviewer comment

The English language should be carefully revised to improve grammar, sentence structure, and overall readability.

**Response**

We sincerely thank the reviewer for this valuable recommendation. In response, the entire manuscript has undergone a comprehensive revision of the English language to improve grammar, sentence structure, clarity, consistency, and overall readability. Particular attention was given to the Introduction and Discussion sections, where several paragraphs were reorganized, redundant expressions were removed, and scientific terminology was standardized. These revisions were made to enhance the overall quality of the manuscript and facilitate communication of the study's findings.

**Changes made in the manuscript**

The entire manuscript was carefully edited to improve English grammar, sentence structure, consistency of terminology, and overall readability. Several sections were rewritten for greater clarity and conciseness while preserving the original scientific content.

**End of Reviewer #1**

We once again sincerely thank Reviewer #1 for the careful evaluation of our manuscript and for the constructive suggestions, which have substantially improved the scientific quality, clarity, and presentation of this work.

**Reviewer #2**

We sincerely thank Reviewer #2 for the thorough and constructive evaluation of our manuscript. We greatly appreciate the detailed comments and thoughtful suggestions, which have significantly contributed to improving the scientific quality, methodological rigor, and overall presentation of our work. In response to the reviewer's recommendations, we carefully revised the manuscript by strengthening the statistical analyses, refining the economic optimization framework, improving the interpretation of the results, updating the literature, and enhancing the clarity and organization of the manuscript. Below, we provide a detailed point-by-point response to each comment.

**Comment 1**

Reviewer comment

The title should better reflect the scope of the study and indicate where the research was conducted.

**Response**

We sincerely thank the reviewer for this valuable suggestion. We agree that the title should clearly reflect both the scope of the study and its geographical context. Accordingly, we revised the title to explicitly indicate that the study focuses on the bioeconomic optimization of zinc and iron agronomic biofortification in cowpea under tropical field conditions in Mozambique. The revised title more accurately represents the objectives, methodology, and regional relevance of the study.

**Changes made in the manuscript**

The manuscript title was revised as follows:

Bioeconomic optimization of zinc and iron agronomic biofortification in cowpea (Vigna unguiculata [L.] Walp.) under tropical field conditions in Mozambique.

**Comment 2**

Reviewer comment

The Abstract should more clearly state the research gap, novelty, and practical significance of the study.

**Response**

We sincerely thank the reviewer for this valuable suggestion. We agree that the Abstract should more clearly highlight the scientific rationale, novelty, and practical significance of the study. Accordingly, the Abstract was comprehensively revised to better define the research gap by emphasizing the limited availability of bioeconomic recommendations for Zn and Fe fertilization in cowpea under tropical conditions. The novelty of integrating agronomic and economic optimization through Response Surface Methodology (RSM) was explicitly highlighted. We also strengthened the concluding statements to emphasize the identification of biologically and economically efficient fertilization strategies capable of maintaining high productivity while improving farm profitability under the agronomic, environmental, and economic conditions evaluated in this study.

**Changes made in the manuscript**

The Abstract was substantially revised to:

• Clearly define the research gap addressed by the study.

• Explicitly highlight the novelty of integrating agronomic and economic optimization using Response Surface Methodology.

• Strengthen the practical implications of the findings by emphasizing biologically and economically efficient fertilization strategies under the conditions evaluated.

• Improve the overall clarity, logical flow, and readability of the Abstract.

**Comment 3**

Reviewer comment

The Introduction should better justify the novelty of the study and clearly explain the knowledge gap addressed by the research.

**Response**

We sincerely thank the reviewer for this valuable comment. We agree that the scientific rationale and novelty of the study should be more clearly presented. Accordingly, we substantially revised the Introduction to better define the existing knowledge gap regarding the integration of agronomic and economic optimization of Zn and Fe fertilization in cowpea under tropical field conditions. We also strengthened the justification for the study by emphasizing that, although the agronomic benefits of Zn and Fe fertilization have been widely documented, fertilizer recommendations are still predominantly based on maximizing biological yield, with limited consideration of economic optimization. Furthermore, the Introduction now explicitly highlights the novelty of integrating Response Surface Methodology (RSM) with bioeconomic analysis to identify fertilization strategies that maximize both grain productivity and farm profitability under the conditions of Mozambique.

**Changes made in the manuscript**

The Introduction was substantially revised to:

Clearly define the scientific knowledge gap addressed by the study.

Strengthen the justification for integrating agronomic and economic optimization.

Highlight the novelty of applying Response Surface Methodology (RSM) to identify both the Maximum Technical Efficiency (MTE) and Maximum Economic Efficiency (MEE).

Emphasize the relevance of the study for developing economically viable micronutrient management strategies for cowpea production in Mozambique and other tropical production systems.

**Comment 4**

Reviewer comment

The Introduction contains several general statements that should be supported by more recent and directly relevant references.

**Response**

We sincerely thank the reviewer for this valuable suggestion. We agree that the Introduction should be supported by recent and directly relevant literature. Accordingly, we thoroughly revised the Introduction by updating the reference list and replacing several older or less specific citations with recent publications that more directly support the statements presented. Particular emphasis was placed on recent studies addressing agronomic biofortification, micronutrient management, nutrient-use efficiency, and bioeconomic optimization under tropical production systems. These revisions strengthen the scientific foundation of the Introduction and ensure that the discussion is supported by current evidence.

**Changes made in the manuscript**

The Introduction was comprehensively updated by:

Replacing outdated or less relevant references with recent publications.

Incorporating additional literature on agronomic biofortification, Zn and Fe nutrition, nutrient-use efficiency, and bioeconomic optimization.

Ensuring that each scientific statement is supported by directly relevant and up-to-date references.

Improving the overall coherence and scientific foundation of the Introduction.

**Comment 5**

Reviewer comment

The Materials and Methods section should provide a clearer description of the experimental design, statistical analysis, and optimization procedures.

**Response**

We sincerely thank the reviewer for this valuable comment. We agree that a clearer description of the experimental design and statistical procedures improves the transparency and reproducibility of the study. Accordingly, we substantially revised the Materials and Methods section to provide a more comprehensive description of the experimental design, the factorial analysis of variance (ANOVA), the response surface methodology (RSM), and the procedures used to estimate the Maximum Technical Efficiency (MTE) and Maximum Economic Efficiency (MEE). We also clarified the sequence of the statistical analyses, including model assumptions, response surface modeling, economic optimization, and agronomic efficiency calculations. These revisions provide a more complete and transparent description of the analytical framework adopted in the study.

**Changes made in the manuscript**

The Materials and Methods section was substantially revised to:

Clarify the experimental design and factorial treatment structure.

Improve the description of the factorial ANOVA and statistical model.

Clearly describe the response surface methodology (RSM) used for agronomic and economic optimization.

Explain the procedures used to estimate the Maximum Technical Efficiency (MTE) and Maximum Economic Efficiency (MEE).

Reorganize the statistical analysis to improve clarity, transparency, and reproducibility.

**Comment 6**

Reviewer comment

The rationale for using Response Surface Methodology (RSM) should be better explained and justified.

**Response**

We sincerely thank the reviewer for this valuable suggestion. We agree that the rationale for using Response Surface Methodology (RSM) should be more clearly explained. Accordingly, we revised the Materials and Methods section to better justify the use of RSM as an appropriate analytical approach for simultaneously evaluating the individual and interactive effects of Zn and Fe fertilization, describing nonlinear crop responses, and identifying biologically and economically optimal fertilizer combinations. We also clarified that RSM was selected because it enables the estimation of both the Maximum Technical Efficiency (MTE) and the Maximum Economic Efficiency (MEE), thereby providing a robust framework for integrating agronomic and economic decision-making.

**Changes made in the manuscript**

The Materials and Methods section was revised to strengthen the justification for the use of Response Surface Methodology (RSM). The revised text explains its suitability for modeling nonlinear responses, evaluating nutrient interactions, and identifying both agronomic and economic optimum fertilizer rates, thereby improving the transparency and scientific rationale of the analytical approach adopted in the study.

**Comment 7**

Reviewer comment

The statistical results should be presented more clearly, particularly the factorial ANOVA, interaction effects, and the interpretation of the response surface model.

**Response**

We sincerely thank the reviewer for this valuable comment. We agree that a clearer presentation of the statistical results improves the readability and interpretation of the manuscript. Accordingly, we revised the Results section to provide a more systematic description of the factorial ANOVA, clearly distinguishing the main effects and interaction effects. We also improved the interpretation of the response surface model by explicitly discussing the significance of the linear, quadratic, and interaction terms and their contribution to the observed crop responses. In addition, references to the corresponding tables were incorporated throughout the text to facilitate comparison between the narrative description and the statistical results.

**Changes made in the manuscript**

The Results section was substantially revised to:

Present the factorial ANOVA results more clearly and systematically.

Improve the interpretation of the main effects and interaction effects.

Strengthen the explanation of the response surface model, including the linear, quadratic, and interaction terms.

Include direct references to the corresponding tables to improve consistency between the text and the statistical analyses.

**Comment 8**

Reviewer comment

The ANOVA results presented in Table 2 should be more clearly explained in the text, particularly regarding the significance of the interaction terms.

**Response**

We sincerely thank the reviewer for this valuable comment. We agree that a clearer interpretation of the ANOVA results improves the readability of the manuscript. Accordingly, we revised the Results section to provide a more comprehensive explanation of the factorial ANOVA, with particular emphasis on the biological interpretation of the significant interaction terms. The revised text now clearly distinguishes the effects of fertilization mode, Zn rates, Fe rates, and their interactions, while also reporting the corresponding P-values and directing readers to **Table 3** for the complete statistical results.

**Changes made in the manuscript**

The Results section was revised to:
Improve the interpretation of the factorial ANOVA.
Clearly explain the biological significance of the interaction terms.
Report the corresponding P-values for the principal interaction effects discussed in the text.
Include direct references to **Table 3**, improving consistency between the narrative description and the statistical analysis.

**Comment 9**

Reviewer comment

The response surface regression model should be presented and interpreted more clearly. Please explain the biological meaning of the regression coefficients and discuss the practical implications of the optimum fertilizer rates.

**Response**

We sincerely thank the reviewer for this valuable comment. We agree that a clearer presentation and interpretation of the response surface model improves the scientific value of the manuscript. Accordingly, we substantially revised the Results section to explicitly present the fitted second-order polynomial equation, together with its coefficient of determination (R²) and adjusted R². We also expanded the interpretation of the regression coefficients by explaining the biological significance of the linear, quadratic, and interaction terms. In addition, the practical implications of the estimated optimum fertilizer rates were strengthened by discussing their relevance for maximizing grain productivity while improving fertilizer-use efficiency under the experimental conditions.

**Changes made in the manuscript**

The Results section was substantially revised to:

Present the fitted second-order polynomial regression equation.

Report the coefficient of determination (R²) and adjusted R².

Provide a clearer biological interpretation of the linear, quadratic, and interaction coefficients.

Expand the discussion of the practical significance of the estimated optimum Zn and Fe application rates for agronomic management.

**Comment 10**

Reviewer comment

The economic analysis is interesting; however, the manuscript should better explain how the Maximum Economic Efficiency (MEE) was determined and how it differs from the Maximum Technical Efficiency (MTE).

**Response**

We sincerely thank the reviewer for this valuable comment. We agree that a clearer distinction between Maximum Technical Efficiency (MTE) and Maximum Economic Efficiency (MEE) improves the interpretation of the study. Accordingly, we substantially revised both the Materials and Methods and Discussion sections to clarify the conceptual and practical differences between these optimization criteria. The revised manuscript explains that MTE represents the combination of Zn and Fe application rates that maximizes predicted grain yield, whereas MEE identifies the fertilization strategy that maximizes net economic return by simultaneously considering crop response and production costs. We further emphasize that the economically optimal fertilizer rates may differ from those maximizing biological yield because of diminishing marginal returns and the additional cost associated with fertilizer inputs, particularly Fe-EDTA.

**Changes made in the manuscript**

The manuscript was revised to:

Clearly distinguish the concepts of Maximum Technical Efficiency (MTE) and Maximum Economic Efficiency (MEE). Expand the description of the bioeconomic optimization procedure in the Materials and Methods section. Strengthen the Discussion by explaining why the economically optimal fertilizer rates differed from the agronomic optimum under the conditions of this study. Highlight the practical implications of integrating agronomic and economic criteria for fertilizer recommendation.

**Comment 11**

Reviewer comment

The manuscript should provide a clearer explanation of the Benefit:Cost (B:C) ratio and its relationship with net profit in evaluating the economic performance of the fertilization strategies.

**Response**

We sincerely thank the reviewer for this valuable comment. We agree that the economic indicators used in the study should be clearly distinguished and interpreted. Accordingly, we revised the Materials and Methods, Results, and Discussion sections to better explain the complementary roles of the Benefit:Cost (B:C) ratio and Net Profit (NP) in the economic evaluation. The revised manuscript clarifies that the B:C ratio measures the return obtained per unit of production cost, whereas NP represents the absolute economic return after deducting all production costs. We further emphasize that both indicators provide complementary information and together offer a more comprehensive assessment of the economic viability of the fertilization strategies evaluated.

**Changes made in the manuscript**

The manuscript was revised to:

Clearly define the Benefit:Cost (B:C) ratio and Net Profit (NP).

Explain the conceptual differences between these two economic indicators.

Expand the interpretation of the economic results by emphasizing the complementary roles of B:C ratio and NP in evaluating fertilizer profitability.

Improve the discussion of the practical implications of the economic analysis for fertilizer recommendation.

**Comment 12**

Reviewer comment

The Discussion should be revised to avoid overinterpretation of the results and to ensure that conclusions are fully supported by the experimental evidence.

**Response**

We sincerely thank the reviewer for this valuable and constructive comment. We fully agree that the interpretation of the results should remain within the scope of the experimental evidence. Accordingly, we comprehensively revised the Discussion to moderate several statements that could be interpreted as causal or overly generalized. Physiological mechanisms underlying the observed responses are now consistently presented as plausible explanations supported by previous literature rather than as direct evidence from the present study. In addition, we explicitly acknowledged the limitations associated with the single-site, single-season experiment and avoided extending the conclusions beyond the environmental and management conditions evaluated.

**Changes made in the manuscript**

The Discussion was substantially revised to:

Moderate interpretations that were not directly supported by the experimental evidence.

Present physiological mechanisms as plausible explanations supported by the literature rather than definitive conclusions.

Explicitly acknowledge the limitations of a single-site, single-season experiment.

Improve the overall balance between the experimental findings and their interpretation.

**Comment 13**

Reviewer comment

The Conclusion should more clearly reflect the limitations of the study and avoid making recommendations beyond the scope of the experimental evidence.

**Response**

We sincerely thank the reviewer for this valuable comment. We fully agree that the conclusions should accurately reflect the scope and limitations of the study. Accordingly, we revised the Conclusion to ensure that all statements are directly supported by the experimental findings. The revised conclusion now emphasizes that the identified agronomic and economic optimum fertilizer rates are applicable to the environmental and management conditions under which the study was conducted. We also explicitly acknowledge that additional multi-location and multi-season experiments are required to validate these recommendations across different agroecological conditions before they can be generalized.

**Changes made in the manuscript**

The Conclusion section was revised to:

Moderate statements that could be interpreted as overly general.

Explicitly acknowledge the limitations associated with a single-site and single-season field experiment.

Clarify the scope of applicability of the findings.

Recommend future multi-location and multi-season studies to validate the proposed fertilizer recommendations under different agroecological conditions.

**Comment 14**

Reviewer comment

The figures and tables should be improved to enhance readability and facilitate interpretation of the results.

**Response**

We sincerely thank the reviewer for this valuable suggestion. We agree that clear presentation of figures and tables is essential for effective communication of the results. Accordingly, all figures and tables were carefully revised to improve their readability, consistency, and overall presentation. Figure quality was enhanced by increasing image resolution, standardizing font sizes and labels, and improving the clarity of legends. The tables were reformatted to improve organization and readability, and their captions were revised to provide clearer descriptions of the information presented.

**Changes made in the manuscript**

The figures and tables were comprehensively revised to improve visual quality, formatting consistency, and readability. Figure legends and table captions were also edited to provide clearer descriptions and facilitate interpretation of the results

Once again, we sincerely thank the Academic Editor and both reviewers for their careful evaluation and constructive suggestions. Their comments have substantially improved the scientific quality, clarity, and overall presentation of the manuscript. We hope that the revisions have satisfactorily addressed all concerns and respectfully submit the revised manuscript for your reconsideration.

Sincerely,

**Tancredo José Carlos**

On behalf of all authors
