# Supplementary material for: Bioeconomic optimization of zinc and iron agronomic biofortification in cowpea (Vigna unguiculata [L.] Walp.) in Mozambique under tropical field conditions
Source: PLoS One. 2026 Sep 3;21(9):e0357473. doi: 10.1371/journal.pone.0357473 (PMC13541262; doi:10.1371/journal.pone.0357473)
Supplement: Peer review history

# Peer review: Decision Letter 0

**DOI:** 10.1371/journal.pone.0357473.r001

**Authors:** Muhammad Amjad Ali

29 Jul 2026

Dear Dr. Carlos,

Thank you for submitting your manuscript to PLOS ONE. After careful consideration, we feel that it has merit but does not fully meet PLOS ONE’s publication criteria as it currently stands. Therefore, we invite you to submit a revised version of the manuscript that addresses the points raised during the review process.

Please submit your revised manuscript by Sep 27 2026 11:59PM. If you will need more time than this to complete your revisions, please reply to this message or contact the journal office at plosone@plos.org. When you're ready to submit your revision, log on to https://www.editorialmanager.com/pone/ and select the 'Submissions Needing Revision' folder to locate your manuscript file.

- A letter that responds to each point raised by the academic editor and reviewer(s). You should upload this letter as a separate file labeled 'Response to Reviewers'.
- A marked-up copy of your manuscript that highlights changes made to the original version. You should upload this as a separate file labeled 'Revised Manuscript with Track Changes'.
- An unmarked version of your revised paper without tracked changes. You should upload this as a separate file labeled 'Manuscript'.

If you would like to make changes to your financial disclosure, please include your updated statement in your cover letter. Guidelines for resubmitting your figure files are available below the reviewer comments at the end of this letter.

If applicable, we recommend that you deposit your laboratory protocols in protocols.io to enhance the reproducibility of your results. Protocols.io assigns your protocol its own identifier (DOI) so that it can be cited independently in the future. For instructions see: https://journals.plos.org/plosone/s/submission-guidelines#loc-laboratory-protocols. Additionally, PLOS ONE offers an option for publishing peer-reviewed Lab Protocol articles, which describe protocols hosted on protocols.io. Read more information on sharing protocols at https://plos.org/protocols?utm_medium=editorial-email&utm_source=authorletters&utm_campaign=protocols.

As the corresponding author, your ORCID iD is verified in the submission system and will appear in the published article. PLOS supports the use of ORCID, and we encourage all coauthors to register for an ORCID iD and use it as well. Please encourage your coauthors to verify their ORCID iD within the submission system before final acceptance, as unverified ORCID iDs will not appear in the published article. *Only*  the individual author can complete the verification step; PLOS staff *cannot*  verify ORCID iDs on behalf of authors.

We look forward to receiving your revised manuscript.

Kind regards,

Muhammad Amjad Ali, PhD

Academic Editor

PLOS One

Journal Requirements:

When submitting your revision, we need you to address these additional requirements.

1. Please ensure that your manuscript meets PLOS ONE's style requirements, including those for file naming. The PLOS ONE style templates can be found at https://journals.plos.org/plosone/s/file?id=wjVg/PLOSOne_formatting_sample_main_body.pdf and https://journals.plos.org/plosone/s/file?id=ba62/PLOSOne_formatting_sample_title_authors_affiliations.pdf

2. We note that the grant information you provided in the ‘Funding Information’ and ‘Financial Disclosure’ sections do not match.

When you resubmit, please ensure that you provide the correct grant numbers for the awards you received for your study in the ‘Funding Information’ section.

3. Thank you for stating the following financial disclosure:

[Regional Scholarship and Innovation Fund (RSIF), (B8501N10019), Mr Tancredo Jose Carlos; -Partnership for Skills in Applied Sciences, Engineering, and Technology (PASET), I ampleased to inform you that, following a highly competitive selection process, you have been selected to receive a Regional Scholarship and Innovation Fund (RSIF),].

Please state what role the funders took in the study.  If the funders had no role, please state: "The funders had no role in study design, data collection and analysis, decision to publish, or preparation of the manuscript."

If this statement is not correct you must amend it as needed.

Please include this amended Role of Funder statement in your cover letter; we will change the online submission form on your behalf.

4. In the online submission form, you indicated that [All relevant data underlying the results presented in this study are available from the corresponding author upon reasonable request. All summarized results generated from the dataset are included within the manuscript.].

All PLOS journals now require all data underlying the findings described in their manuscript to be freely available to other researchers, either 1. In a public repository, 2. Within the manuscript itself, or 3. Uploaded as supplementary information.

This policy applies to all data except where public deposition would breach compliance with the protocol approved by your research ethics board. If your data cannot be made publicly available for ethical or legal reasons (e.g., public availability would compromise patient privacy), please explain your reasons on resubmission and your exemption request will be escalated for approval.

5. Please upload a new copy of Figure 1 as the detail is not clear. Please follow the link for more information:  https://journals.plos.org/plosone/s/figures

6. We note that Figure 1 in your submission contains map images which may be copyrighted. All PLOS content is published under the Creative Commons Attribution License (CC BY 4.0), which means that the manuscript, images, and Supporting Information files will be freely available online, and any third party is permitted to access, download, copy, distribute, and use these materials in any way, even commercially, with proper attribution. For these reasons, we cannot publish previously copyrighted maps or satellite images created using proprietary data, such as Google software (Google Maps, Street View, and Earth). For more information, see our copyright guidelines: http://journals.plos.org/plosone/s/licenses-and-copyright.

We require you to either (1) present written permission from the copyright holder to publish these figures specifically under the CC BY 4.0 license, or (2) remove the figures from your submission:

1. You may seek permission from the original copyright holder of Figure 1 to publish the content specifically under the CC BY 4.0 license.

We recommend that you contact the original copyright holder with the Content Permission Form (http://journals.plos.org/plosone/s/file?id=7c09/content-permission-form.pdf) and the following text:

“I request permission for the open-access journal PLOS ONE to publish XXX under the Creative Commons Attribution License (CCAL) CC BY 4.0 (http://creativecommons.org/licenses/by/4.0/). Please be aware that this license allows unrestricted use and distribution, even commercially, by third parties. Please reply and provide explicit written permission to publish XXX under a CC BY license and complete the attached form.”

Please upload the completed Content Permission Form or other proof of granted permissions as an "Other" file with your submission.

In the figure caption of the copyrighted figure, please include the following text: “Reprinted from [ref] under a CC BY license, with permission from [name of publisher], original copyright [original copyright year].”

2. If you are unable to obtain permission from the original copyright holder to publish these figures under the CC BY 4.0 license or if the copyright holder’s requirements are incompatible with the CC BY 4.0 license, please either i) remove the figure or ii) supply a replacement figure that complies with the CC BY 4.0 license. Please check copyright information on all replacement figures and update the figure caption with source information. If applicable, please specify in the figure caption text when a figure is similar but not identical to the original image and is therefore for illustrative purposes only.

The following resources for replacing copyrighted map figures may be helpful:

USGS National Map Viewer (public domain): http://viewer.nationalmap.gov/viewer/

The Gateway to Astronaut Photography of Earth (public domain): http://eol.jsc.nasa.gov/sseop/clickmap/

Maps at the CIA (public domain): https://www.cia.gov/library/publications/the-world-factbook/index.html and https://www.cia.gov/library/publications/cia-maps-publications/index.html

NASA Earth Observatory (public domain): http://earthobservatory.nasa.gov/

Landsat: http://landsat.visibleearth.nasa.gov/

USGS EROS (Earth Resources Observatory and Science (EROS) Center) (public domain): http://eros.usgs.gov/#

Natural Earth (public domain): http://www.naturalearthdata.com/

7. We note you have included a table to which you do not refer in the text of your manuscript. Please ensure that you refer to Table 2 in your text; if accepted, production will need this reference to link the reader to the Table.

If the reviewer comments include a recommendation to cite specific previously published works, please review and evaluate these publications to determine whether they are relevant and should be cited. There is no requirement to cite these works unless the editor has indicated otherwise.

[Note: HTML markup is below. Please do not edit.]

Reviewers' comments:

Reviewer's Responses to Questions

**Comments to the Author**

1. Is the manuscript technically sound, and do the data support the conclusions?

Reviewer #1: Yes

Reviewer #2: Yes

**********

2. Has the statistical analysis been performed appropriately and rigorously?

Reviewer #1: Yes

Reviewer #2: Yes

**********

3. Have the authors made all data underlying the findings in their manuscript fully available?

The PLOS Data policy

Reviewer #1: Yes

Reviewer #2: Yes

**********

4. Is the manuscript presented in an intelligible fashion and written in standard English?

Reviewer #1: Yes

Reviewer #2: Yes

**********

Reviewer #1: Dear All,

The manuscript evaluates the agronomic and economic optimization of zinc (Zn) and iron (Fe) fertilization for cowpea biofortification under tropical field conditions. Using response surface methodology, the authors investigated the effects of different Zn and Fe application rates and methods on grain yield, grain Zn and Fe concentrations, and economic return. The study demonstrates that the combined management of Zn and Fe improves crop productivity, nutritional quality, and profitability, allowing the identification of fertilization strategies that optimize both agronomic and economic performance. The topic is timely and relevant because agronomic biofortification has become an important strategy to alleviate micronutrient deficiencies while maintaining crop productivity. Overall, the manuscript presents valuable field data and an appropriate experimental approach. Nevertheless, several physiological interpretations require further refinement, and some conclusions should be moderated because they extend beyond the direct evidence generated by the study.

* Strong points *

1) The manuscript addresses a highly relevant issue concerning agronomic biofortification and food security.

2) The study combines agronomic, nutritional, and economic evaluations, increasing its practical significance.

3) The field experiment provides information obtained under realistic production conditions.

4) Response Surface Methodology is appropriate for optimizing fertilization strategies.

5) The manuscript is logically organized and generally well written.

6) The practical recommendations are potentially useful for tropical cowpea production systems.

* Weaker aspects *

1) Several physiological explanations regarding Zn–Fe interactions are presented as demonstrated mechanisms rather than plausible interpretations.

2) The distinction between nutrient accumulation and nutrient use efficiency deserves further clarification.

3) Soil processes influencing Zn and Fe availability are only briefly discussed.

4) The limitations associated with evaluating a single growing season should be more explicitly acknowledged.

5) The Discussion would benefit from a broader integration of plant nutrition concepts with the bioeconomic analysis.

So, I have annotated below an attempt to clarify certain ideas, but the authors should examine my suggested wording changes carefully, to be sure that I have not misinterpreted what they wanted to say.

1. The manuscript concludes that the combined application of Zn and Fe promotes synergistic effects that improve grain yield and biofortification. Although the response surface analysis clearly demonstrates positive interactions between the two nutrients, the physiological mechanisms responsible for these responses were not directly investigated. Therefore, the Discussion should distinguish more carefully between statistically detected synergistic responses and the underlying physiological mechanisms, which remain hypothetical under the present experimental conditions.

2. Throughout the Discussion, increased grain Zn and Fe concentrations are frequently interpreted as evidence of improved nutrient uptake efficiency. However, grain nutrient accumulation reflects the combined effects of nutrient acquisition, internal redistribution, remobilization during grain filling, and biomass production. Consequently, the Discussion should avoid equating increased grain concentration exclusively with greater uptake efficiency.

3. The bioeconomic optimization is one of the principal strengths of the manuscript. Nevertheless, the economic recommendations should acknowledge that optimum fertilizer rates may vary according to fertilizer prices, grain market values, soil fertility, and regional production systems. A brief discussion of these limitations would improve the applicability of the recommendations.

4. The interaction between Zn and Fe is discussed mainly from the perspective of plant physiology. However, soil chemical processes—including adsorption, precipitation, pH effects, and competition for sorption sites—also regulate the availability of these micronutrients. Incorporating this broader soil–plant perspective would strengthen the interpretation of the observed responses.

5. The experiment was conducted during a single cropping season at one experimental location. While the results are promising, the Conclusions should recognize that additional validation across contrasting soil types, climatic conditions, and production environments is necessary before recommending generalized fertilization strategies.

- Specific comments -

P1 L20–26: The abstract concludes that the optimized Zn–Fe fertilization strategy improves both productivity and profitability. I suggest indicating that these conclusions are valid under the environmental and economic conditions evaluated in this study, avoiding broader generalization.

P1 L30–36: The term "bioeconomic optimization" is central to the manuscript. Consider briefly defining this concept in the Abstract so that readers unfamiliar with the approach immediately understand that both biological and economic responses were simultaneously optimized.

P3 L115–128 (Soil Analysis): The authors provide a useful baseline characterization of the experimental soil, including texture class (sandy-loam), pH, organic carbon, available P, exchangeable K, DTPA-extractable Zn and Fe, and the analytical methods used (Table 1). These data are appropriate for interpreting micronutrient biofortification responses. However, the manuscript does not identify the soil according to an internationally recognized soil classification system (e.g., WRB or USDA Soil Taxonomy). Because soil class strongly influences Zn and Fe availability through differences in mineralogy, buffering capacity, drainage, and sorption processes, I recommend that the authors report the formal soil classification together with the corresponding reference.

P4 L92–99: Please report whether the experimental season was climatically representative of the long-term average for the region. This information would help readers evaluate the broader applicability of the proposed fertilization strategy.

P4 L100–108: Include the soil classification (WRB or USDA Soil Taxonomy), since soil class strongly influences Zn and Fe availability and facilitates comparison with similar field studies.

P4 L109–117: Please clarify whether DTPA-extractable Zn and Fe were determined before fertilizer application. These values are important for interpreting crop responsiveness to micronutrient fertilization.

P5 L118–127: Indicate whether the micronutrient fertilizers were surface-applied or incorporated into the soil, as fertilizer placement may influence Zn and Fe availability.

P5 L130–138: Please specify whether soil moisture was monitored during the growing season, since water availability directly affects micronutrient diffusion and plant uptake.

P6 L145–153: The description of the experimental design is clear. Nevertheless, briefly indicate whether possible border effects were minimized between treatment plots.

P6 L160–169: Clarify whether grain yield was corrected to a standard moisture content before statistical analysis.

P7 L176–186: The response surface methodology is appropriate. However, please indicate whether residual analyses confirmed the assumptions of normality and homogeneity of variance.

P8 L200–208: The manuscript reports significant Zn × Fe interactions. Please provide the corresponding interaction P-values within the text or refer readers directly to the ANOVA table.

P9 L218–226: Consider reporting confidence intervals for the predicted optimum nutrient rates, which would provide an indication of the robustness of the optimization model.

P10 L236–245: The response surfaces are informative. Adding contour values or predicted yield labels would facilitate interpretation of the biological optimum.

P11 L252–260: The grain Zn and Fe concentrations increased substantially. Please discuss whether dilution effects associated with higher grain yield were considered during data interpretation.

P12 L270–279: The manuscript attributes increased grain micronutrient concentration to improved nutrient uptake. This interpretation should be moderated because internal remobilization during grain filling was not evaluated.

P13 L285–294: Please clarify whether nutrient accumulation was calculated on a concentration basis or total nutrient content per unit area, since these parameters provide different physiological information.

P14 L300–308: The economic analysis is one of the strengths of the manuscript. Nevertheless, indicate the fertilizer prices and grain market prices used, together with their reference period.

P15 L309-317: It would be useful to mention whether sensitivity analyses were conducted for different fertilizer price scenarios.

P20 L313-316: The Discussion appropriately emphasizes that plant performance depends on nutrient balance rather than the absolute availability of Zn or Fe. This section could be strengthened by highlighting that plant nutritional responses arise from integrated interactions among nutrient acquisition, soil processes, and plant physiology rather than from isolated nutrient effects.

NOTICE: Suggested insertion: "This observation reinforces the concept that plant nutritional performance depends on integrated interactions among nutrient acquisition, soil processes, and physiological regulation, rather than on the isolated availability of individual nutrients (Thiengo et al., 2026 - https://doi.org/10.1590/1678-992X-2026-0067 )."

P20 L313-321: The authors discuss the increase in grain Zn concentration following micronutrient fertilization. This discussion would benefit from acknowledging that the effectiveness of agronomic Zn biofortification depends not only on fertilizer supply but also on the timing of application and genotype-specific responses.

NOTICE: Suggested insertion: "Previous studies have demonstrated that successful agronomic Zn biofortification depends on the interaction between application timing and genotype, reinforcing that crop responses to foliar Zn fertilization are strongly influenced by physiological differences among cultivars (Morais et al, 2025 - https://doi.org/10.1590/1678-992X-2024-0191."

P21 L318–325: The discussion of Zn × Fe synergism is appropriate. However, the physiological mechanisms should be presented as plausible explanations because root architecture, transporter activity, and gene expression were not evaluated.

P21 L322–326: The statement that Zn improves Fe acquisition through enhanced root development should be interpreted cautiously, since root morphological traits were not measured.

P21 L324–330: Consider discussing the contribution of root-induced rhizosphere modifications to Zn and Fe acquisition, while acknowledging that these processes were not directly investigated.

P21 L330–337: The distinction between nutrient acquisition efficiency and nutrient utilization efficiency should be clarified because these represent different physiological processes.

P21 L338–343: Increased grain micronutrient concentration does not necessarily indicate greater uptake efficiency. Redistribution during grain filling may also contribute to the observed responses.

P22 L344–350: The discussion of diminishing marginal returns is well presented. Nevertheless, indicate that the economic optimum may change with fluctuations in fertilizer prices and grain value.

P22 L346–350: The recommendation for reduced Fe application should be presented within the context of the specific soil fertility conditions evaluated in this experiment.

P22 L351–360: The uncertainty analysis strengthens the manuscript. Please clarify whether climatic variability between years was incorporated into the economic simulations.

P22 L361–370: Consider discussing whether genotype × environment interactions may influence the robustness of the proposed optimum fertilization strategy.

P23 L373–380: The Conclusions appropriately summarize the findings. However, the statement that Zn deficiency strongly constrained productivity should be limited to the experimental conditions evaluated.

P23 L378–384: Please indicate that the optimized fertilization rates should be validated under contrasting soil types before broad recommendations are proposed.

P23 L384–390: The manuscript would benefit from mentioning future research integrating micronutrient fertilization with genotype selection for biofortification.

Reviewer #2: Overview

Manuscript: Bioeconomic optimization of zinc and iron agronomic biofortification in cowpea (Vigna unguiculata [L.] Walp.) under tropical field conditions

Manuscript ID: PONE-D-26-24166

Recommendation: Major revision

The study addresses an important topic and the comparison between yield-maximizing and profit-maximizing fertilizer rates could be valuable. The factorial field experiment also appears to have generated a substantial dataset. However, the manuscript currently contains several conceptual, experimental, statistical, and economic inconsistencies that prevent the main conclusions from being accepted. Nevertheless, the absence of grain micronutrient measurements, ambiguity concerning treatment doses, incorrect or insufficiently reported statistical analysis, pooling of distinct application modes, and use of an average application cost undermine the central conclusions. A substantial reanalysis and major revisions are required before the manuscript can be considered scientifically reliable.

Comments

1.The title should include Mozambique and indicate that this was a single-season field study. If grain mineral concentrations were not measured, “biofortification” should be removed from the title.

2.The Abstract should specify whether rates refer to elemental nutrients or fertilizer products.

3.“Kg” should be written as “kg” throughout.

4.Use consistent formatting for ha⁻¹, mg kg⁻¹, USD ha⁻¹ and percentages.

5.Avoid unnecessary capitalization of terms such as “Maximum Technical Efficiency” within running text.

6.The statement that Fe application continued to increase yield at high rates should be checked against the fitted surface, which ultimately predicts declining yield.

7.The references should be checked for relevance and completeness. Some citations do not directly support the economic claims, several entries are incompletely formatted, and Sheahan and Barrett appears to be duplicated as References 25 and 48.

8.Table 1: Correct inconsistent units for N and P and distinguish measured values from interpretation thresholds. “Physical properties” and “chemical properties” should not be included as empty data rows.

9.Table 2: Degrees of freedom are inconsistent with a four-level factorial analysis. Report sums of squares, mean squares, F statistics and the correct error structure.

10.The missing table should be added or all later tables renumbered.

11.Figures: The figures has low resolution and several labels are difficult to read.

12.Figures 8 and 9: These economic surfaces are based on an average application cost and therefore do not represent a specific application strategy. They should be replaced with mode-specific economic surfaces and uncertainty regions.

13.Figure 12: Define the baseline net profit, the ±20% perturbation procedure, the low and high values used for every variable, and whether variables were changed independently. Numerical outcomes should be included in the figure or a supplementary table.

14.The Introduction and Discussion would benefit from incorporating recent literature that is directly relevant to integrated Zn and Fe fertilization, crop physiological responses, grain micronutrient bioavailability, and the nutritional importance of these elements. In particular, the authors may consider citing Alkarawi (2025) when discussing the effects of combined Fe- and Zn-based formulations on legume growth and yield; Younas et al. (2025) in relation to integrated fertilization and grain Fe bioavailability; Muhae-Ud-Din et al. (2026) when discussing metal-mediated changes in ROS metabolism, nitrogen metabolism, yield, and seed quality; and Iftikhar et al. (2024) to support the broader nutritional significance of Fe and Zn.

Alkarawi HH. The effect of integrating nano-formulated iron and zinc chelates and Biohealth® bio-fertilizer on some vegetative growth and yield characteristics of soybean (Glycine max [L.] Merrill). Pak J Agric Sci. 2025;62(4):717–728. doi:10.21162/PAKJAS/25.964.

Younas, A., Farooq, M., Waqar, S., Ghafoor, I., Makhdoom, M., Parveen, A., Kashif, M., & Uzair, M. (2025). Improving grain iron bioavailability in bread wheat through integrated nitrogen and iron fertilization. Integrative Plant Biotechnology, 3(4), 343–350. https://doi.org/10.58257/pbiotech.v3i4.1680

Muhae-Ud-Din G, Zhong F, Jabran M, Wang Y, Smagghe G, Sun X, et al. Metal nanoparticles enhance ROS scavenging, nitrogen metabolism, and stress-responsive pathways to improve soybean yield and seed quality. Plant Physiology and Biochemistry. 2026:111363.

Iftikhar H, Israr B, Butt MS, Pasha I. The impact of iron- and zinc-fortified snacks on cognitive performance in pre-adolescents. Pak J Agric Sci. 2024;61(4):1075–1083. doi:10.21162/PAKJAS/24.360.

15.Conclusions and recommendations are too broad for a single site-year experiment. Please revise.

16.The manuscript would benefit from professional English-language and formatting revision after the scientific and analytical issues have been resolved.

**********

what does this mean?). If published, this will include your full peer review and any attached files.

If you choose “no”, your identity will remain anonymous but your review may still be made public.

**Do you want your identity to be public for this peer review?**  For information about this choice, including consent withdrawal, please see our Privacy Policy

Reviewer #1: No

Reviewer #2: No

**********

[NOTE: If reviewer comments were submitted as an attachment file, they will be attached to this email and accessible via the submission site. Please log into your account, locate the manuscript record, and check for the action link "View Attachments". If this link does not appear, there are no attachment files.]

To ensure your figures meet our technical requirements, please review our figure guidelines: https://journals.plos.org/plosone/s/figures

You may also use PLOS’s free figure tool, NAAS, to help you prepare publication quality figures: https://journals.plos.org/plosone/s/figures#loc-tools-for-figure-preparation.

NAAS will assess whether your figures meet our technical requirements by comparing each figure against our figure specifications.

# Author comment: Author response to Decision Letter 1

**DOI:** 10.1371/journal.pone.0357473.r002

14 Aug 2026

Response to the Academic Editor and Reviewers

Manuscript ID: PONE-D-26-24166

Title:

Bioeconomic optimization of zinc and iron agronomic biofortification in cowpea (Vigna unguiculata [L.] Walp.) under tropical field conditions in Mozambique

Dear Academic Editor and Reviewers,

We sincerely thank the Academic Editor and both reviewers for their careful evaluation of our manuscript and for their constructive comments and insightful suggestions. We greatly appreciate the time and effort invested in reviewing our work, as well as the recognition of its scientific relevance, technical soundness, experimental design, and practical importance.

In response to the comments received, we have thoroughly revised the manuscript. The major revisions include clarification of the experimental design and statistical methodology, complete reanalysis of the factorial analysis of variance (ANOVA), revision of the bioeconomic optimization framework, improvement of the soil and climatic characterization, moderation of physiological interpretations, acknowledgment of the limitations associated with a single-site and single-season experiment, incorporation of recent and directly relevant literature, revision of the figures and tables, and comprehensive editing of the English language and manuscript formatting.

We have carefully addressed every comment raised by the reviewers. Below, each reviewer comment is reproduced in bold, followed by our detailed response and a summary of the corresponding revisions made to the manuscript. All modifications are highlighted in the revised manuscript submitted with tracked changes.

We hope that the revisions have satisfactorily addressed all concerns and have substantially improved the quality, clarity, and scientific rigor of the manuscript.

Sincerely,

Tancredo José Carlos

On behalf of all authors

Responses to Journal Requirements

Journal Requirement 1

Comment:

Please ensure that your manuscript meets PLOS ONE’s style requirements, including those for file naming.

Response:

We sincerely thank the journal for this reminder. The revised manuscript has been carefully reviewed and reformatted to comply with the PLOS ONE style requirements. Terminology, units, abbreviations, table and figure presentation, reference formatting, and file naming have been standardized throughout the submission.

Changes made:

The manuscript formatting was revised according to the PLOS ONE guidelines.

Units, abbreviations, tables, figures, and references were standardized.

The revised files will be uploaded using the required designations: Manuscript, Revised Manuscript with Track Changes, and Response to Reviewers.

Journal Requirement 2

Comment:

The grant information provided in the “Funding Information” and “Financial Disclosure” sections does not match. Please provide the correct grant numbers.

Response:

We sincerely thank the journal for identifying this inconsistency. The funding information has been reviewed and corrected to ensure that the grant number and funding organization are reported consistently in the manuscript, cover letter, and online submission system.

Revised funding statement:

This study was supported by the Regional Scholarship and Innovation Fund (RSIF), a program of the Partnership for Skills in Applied Sciences, Engineering and Technology (PASET), under grant number B8501N10019 awarded to Tancredo José Carlos.

Changes made:

The Funding Information and Financial Disclosure statements were harmonized to report the same funding organization and grant number.

Journal Requirement 3

Comment:

Please state what role the funders took in the study. If the funders had no role, please state: “The funders had no role in study design, data collection and analysis, decision to publish, or preparation of the manuscript.”

Response:

We confirm that the funders had no role in the scientific design, execution, analysis, interpretation, or publication decisions associated with this study.

Revised Role of Funder statement:

The funders had no role in study design, data collection and analysis, decision to publish, or preparation of the manuscript.

Changes made:

This statement has been included in the revised manuscript and will also be provided in the revised online submission information to ensure consistency between the manuscript and the submission system.

Journal Requirement 4

Comment:

All data underlying the findings must be freely available through a public repository, within the manuscript, or as Supporting Information.

Response:

We sincerely thank the journal for this clarification. The data underlying the findings of this study will be made freely available as Supporting Information accompanying the revised manuscript. The dataset includes the experimental factors, block structure, fertilization modes, Zn and Fe application rates, grain yield, and the variables used in the agronomic and economic analyses.

Revised Data Availability statement:

All data underlying the findings reported in this study are provided in the Supporting Information files accompanying the manuscript.

Changes made:

The underlying experimental dataset will be uploaded as Supporting Information.

The Data Availability statement has been revised accordingly.

Journal Requirement 5

Comment:

Please upload a new copy of Figure 1 because the details are not clear.

Response:

We sincerely thank the journal for this comment. Figure 1 has been replaced with a higher-resolution version. Font sizes, labels, geographical boundaries, symbols, and annotations were enlarged and standardized to improve readability.

Changes made:

Figure 1 was regenerated and replaced with a clearer, higher-resolution version that complies with the PLOS ONE figure preparation requirements.

Journal Requirement 6

Comment:

Figure 1 may contain copyrighted map images. Please provide permission or replace the figure with a map that complies with the CC BY 4.0 license.

Response:

We sincerely thank the journal for drawing our attention to this issue. The original map was replaced with a newly generated figure based exclusively on open-access or public-domain spatial data. No Google Maps, Google Earth, proprietary satellite imagery, or other copyrighted map material was used in the revised figure.

Changes made:

Figure 1 was reconstructed using spatial data compatible with open-access publication under the CC BY 4.0 license.

The figure caption was revised to identify the spatial data source.

The previous map containing potentially copyrighted material was removed.

Figure caption source statement:

Administrative boundaries and base-map data were obtained from open-access spatial datasets and the figure was created by the authors

Journal Requirement 7

Comment:

Table 2 is not referred to in the manuscript text. Please ensure that Table 2 is cited in the text.

Response:

We sincerely thank the journal for identifying this omission. In the revised manuscript, the factorial analysis of variance table, originally presented as Table 2, has been renumbered as Table 3 following the inclusion of a new table describing the climatic conditions during the experimental season. The Results section has been revised to refer explicitly to Table 3 when presenting the factorial analysis of variance and the significance of the main effects and interaction terms.

Changes made:

A direct citation to Table 3 was added to the Results section, ensuring that the factorial analysis of variance table is properly linked to the corresponding statistical description in the text.

Reviewer #1

We sincerely thank Reviewer #1 for the thorough evaluation of our manuscript and for the constructive comments and insightful suggestions. We greatly appreciate the reviewer's careful assessment and the positive feedback regarding the scientific quality of our study. The comments have substantially improved the clarity, rigor, and interpretation of the manuscript. We have carefully considered each recommendation and revised the manuscript accordingly. Our point-by-point responses are presented below.

Comment 1

Reviewer comment

Please report the WRB or USDA soil classification of the experimental site.

Response

We sincerely thank the reviewer for this valuable comment. Unfortunately, a site-specific WRB or USDA soil classification was not available for the experimental field. Therefore, we chose not to assign a formal soil classification without direct pedological characterization, as doing so could result in an inaccurate or unsupported classification. Instead, the soil was characterized based on its measured physical and chemical properties obtained before crop establishment, including texture, pH, organic carbon, available phosphorus, exchangeable potassium, sulfur, DTPA-extractable zinc and iron, and total nitrogen. To provide regional pedological context, we also incorporated information from the National Soil Map of Mozambique, which indicates that the study area is predominantly characterized by Arenosols, Ferralsols, Lixisols, and Acrisols. We believe that this approach provides a scientifically sound description of the experimental soil while avoiding unsupported taxonomic classification.

Changes made in the manuscript

The description of the experimental soil in the Materials and Methods section was revised to clarify that the soil was characterized based on its measured physical and chemical properties rather than assigning an unsupported WRB or USDA classification. In addition, regional soil information from the National Soil Map of Mozambique was included to provide pedological context.

Comment 2

Reviewer comment

Please report whether the experimental season was climatically representative of the long-term average for the region.

Response

We sincerely thank the reviewer for this valuable comment. To address this suggestion, we compared the climatic conditions recorded during the 2025 growing season with the long-term climatic normals obtained from the Quelimane Meteorological Station, the closest meteorological station to the experimental site. To facilitate this comparison, we included a new table in the revised manuscript summarizing the observed and long-term climatic conditions. Mean monthly temperatures during the experimental period were very similar to the long-term averages, with only minor differences, while rainfall exhibited some monthly variability but maintained the characteristic seasonal distribution of the region. Overall, this comparison indicates that the experiment was conducted under climatic conditions that were broadly representative of the regional production environment.

Changes made in the manuscript

A new table comparing the climatic conditions observed during the 2025 growing season with the long-term climatic normals for the Quelimane Meteorological Station was added to the manuscript. The Materials and Methods and Results sections were revised accordingly to demonstrate that the experimental season was broadly representative of the climatic conditions of the study region.

Comment 3

Reviewer comment

Please clarify the statistical model used in the analysis and specify whether block was treated as a fixed or random effect.

Response

We sincerely thank the reviewer for this important comment. We carefully revised the Statistical Analysis section to provide a clearer and more accurate description of the statistical model used in this study. The treatment effects were evaluated using a factorial analysis of variance (ANOVA) for a randomized complete block design (RCBD) arranged in a 3 × 4 × 4 factorial scheme, including the effects of block, fertilization mode, Zn rate, Fe rate, and their interactions. Because a mixed-effects model was not fitted, we removed the previous statement indicating that block was treated as a random effect. The statistical model is now described in a manner that accurately reflects the analytical approach adopted in the study.

Changes made in the manuscript

The Statistical Analysis section was revised to provide a clear description of the factorial ANOVA model used in the study. The previous statement indicating that block was treated as a random effect was removed, and the statistical model was rewritten to accurately describe the effects included in the analysis and the experimental design

Comment 4

Reviewer comment

P8 L200–208: The manuscript reports significant Zn × Fe interactions. Please provide the corresponding interaction P-values within the text or refer readers directly to the ANOVA table.

Response

We sincerely thank the reviewer for this helpful suggestion. To improve the transparency and readability of the statistical results, we revised the Results section to explicitly report the corresponding P-values for the interaction terms discussed in the text. In addition, direct references to Table 3 were included wherever the ANOVA results are described, allowing readers to easily relate the narrative interpretation to the complete statistical analysis.

Changes made in the manuscript

The Results section was revised to include the corresponding P-values for the significant interaction terms discussed in the text. Direct references to Table 3 were also added to improve consistency between the narrative description and the ANOVA results.

Comment 5

Reviewer comment

P9 L218–226: Consider reporting confidence intervals for the predicted optimum nutrient rates, which would provide an indication of the robustness of the optimization model.

Response

We sincerely thank the reviewer for this valuable suggestion. We agree that confidence intervals for the estimated optimum nutrient rates could provide additional information regarding the uncertainty associated with the optimization results. However, estimating confidence intervals for the stationary point of a second-order response surface model requires specialized statistical procedures, such as bootstrap resampling or delta-method approaches, which were beyond the scope of the present study. To address the reviewer's concern, we clarified that the estimated optimum occurred within the experimental domain and strengthened the evaluation of model robustness by emphasizing the goodness-of-fit statistics, coefficient of determination (R²), and residual diagnostics used to assess model adequacy.

Changes made in the manuscript

The Statistical Analysis and Results sections were revised to clarify that the estimated optimum was located within the experimental domain. Additional information regarding model adequacy, including the coefficient of determination (R²), residual diagnostics, and model adequacy assessment, was incorporated to provide greater confidence in the robustness of the response surface model.

Comment 6

Reviewer comment

P10 L236–245: The response surfaces are informative. Adding contour values or predicted yield labels would facilitate interpretation of the biological optimum.

Response

We sincerely thank the reviewer for this valuable suggestion. We agree that adding contour labels could further facilitate the interpretation of the response surface. However, the contour plot already clearly identifies the optimum region corresponding to the maximum predicted grain yield. Rather than adding numerical labels that could reduce figure readability, we revised the figure legend to explicitly state that the contour plot represents the predicted grain yield and highlights the region corresponding to the Maximum Technical Efficiency (MTE). We believe this revision improves figure interpretation while preserving its visual clarity.

Changes made in the manuscript

The legend of Fig 5 was revised to clarify that the contour plot represents predicted cowpea grain yield and to explicitly identify the estimated Maximum Technical Efficiency (MTE), including the corresponding Zn and Fe application rates and predicted grain yield. This revision improves interpretation of the biological optimum while preserving the visual clarity of the figure.

Comment 7

Reviewer comment

P11 L252–260: The grain Zn and Fe concentrations increased substantially. Please discuss whether dilution effects associated with higher grain yield were considered during data interpretation.

Response

We sincerely thank the reviewer for this valuable comment. We agree that dilutio

Attachment Submitted filename: Response to the Editor and Reviewers.docx

# Peer review: Decision Letter 1

**DOI:** 10.1371/journal.pone.0357473.r003

**Authors:** Muhammad Amjad Ali

18 Aug 2026

Bioeconomic optimization of zinc and iron agronomic biofortification in cowpea (Vigna unguiculata [L.] Walp.) in Mozambique under tropical field conditions

PONE-D-26-24166R1

Dear Dr. Carlos,

We’re pleased to inform you that your manuscript has been judged scientifically suitable for publication and will be formally accepted for publication once it meets all outstanding technical requirements.

Within one week, you’ll receive an e-mail detailing the required amendments. When these have been addressed, you’ll receive a formal acceptance letter and your manuscript will be scheduled for publication.

An invoice will be generated when your article is formally accepted. Please note, if your institution has a publishing partnership with PLOS and your article meets the relevant criteria, all or part of your publication costs will be covered. Please make sure your user information is up-to-date by logging into Editorial Manager at Editorial Manager® and clicking the ‘Update My Information' link at the top of the page. For questions related to billing, please contact billing support.

If your institution or institutions have a press office, please notify them about your upcoming paper to help maximize its impact. If they’ll be preparing press materials, please inform our press team as soon as possible -- no later than 48 hours after receiving the formal acceptance. Your manuscript will remain under strict press embargo until 2 pm Eastern Time on the date of publication. For more information, please contact onepress@plos.org.

Kind regards,

Muhammad Amjad Ali, PhD

Academic Editor

PLOS One

Additional Editor Comments (optional):

Reviewers' comments:

Reviewer's Responses to Questions

**Comments to the Author**

Reviewer #2: All comments have been addressed

**********

2. Is the manuscript technically sound, and do the data support the conclusions?

Reviewer #2: Yes

**********

3. Has the statistical analysis been performed appropriately and rigorously?

Reviewer #2: Yes

**********

4. Have the authors made all data underlying the findings in their manuscript fully available?

The PLOS Data policy

Reviewer #2: Yes

**********

5. Is the manuscript presented in an intelligible fashion and written in standard English?

Reviewer #2: Yes

**********

Reviewer #2: This is now well-written and executed study. I have no additional comments. I recommend the paper for publication.

**********

what does this mean?). If published, this will include your full peer review and any attached files.

If you choose “no”, your identity will remain anonymous but your review may still be made public.

**Do you want your identity to be public for this peer review?**  For information about this choice, including consent withdrawal, please see our Privacy Policy

Reviewer #2: **Yes:** Muhammad Jabran, PhD.

**********

# Editor report: Acceptance letter

**DOI:** 10.1371/journal.pone.0357473.r004

**Authors:** Muhammad Amjad Ali

PONE-D-26-24166R1

PLOS One

Dear Dr. Carlos,

I'm pleased to inform you that your manuscript has been deemed suitable for publication in PLOS One. Congratulations! Your manuscript is now being handed over to our production team.

At this stage, our production department will prepare your paper for publication. This includes ensuring the following:

* All references, tables, and figures are properly cited

* All relevant supporting information is included in the manuscript submission,

* There are no issues that prevent the paper from being properly typeset

You will receive further instructions from the production team, including instructions on how to review your proof when it is ready. Please keep in mind that we are working through a large volume of accepted articles, so please give us a few days to review your paper and let you know the next and final steps.

Lastly, if your institution or institutions have a press office, please let them know about your upcoming paper now to help maximize its impact. If they'll be preparing press materials, please inform our press team within the next 48 hours. Your manuscript will remain under strict press embargo until 2 pm Eastern Time on the date of publication. For more information, please contact onepress@plos.org.

You will receive an invoice from PLOS for your publication fee after your manuscript has reached the completed accept phase. If you receive an email requesting payment before acceptance or for any other service, this may be a phishing scheme. Learn how to identify phishing emails and protect your accounts at https://explore.plos.org/phishing.

If we can help with anything else, please email us at customercare@plos.org.

Thank you for submitting your work to PLOS One and supporting open access.

Kind regards,

PLOS ONE Editorial Office Staff

on behalf of

Dr. Muhammad Amjad Ali

Academic Editor

PLOS One
